# Proliferative Effects of the Psychedelic *N,N*-Dimethyltryptamine (DMT) in Human Neural Stem Cells

**DOI:** 10.1021/acschemneuro.6c00209

**Published:** 2026-07-10

**Authors:** José Alexandre Salerno, Elizabeth R. Dominguez, Karina Karmirian, Breno A.B.M.S. Arrais, Juliano Alves, Giovanna Erjautz, Kennedy Kroening, Leticia R. Q. Souza, Isis Ornelas, Stevens Rehen

**Affiliations:** † 519983 D’Or Institute for Research and Education (IDOR), Rio de Janeiro, Rio de Janeiro, 22281100, Brazil; ‡ Graduate Program in Morphological Sciences, Institute of Biomedical Sciences, Universidade Federal do Rio de Janeiro (UFRJ), Rio de Janeiro, Rio de Janeiro, 21941599, Brazil; § Department of Morphological Sciences, Biomedical Institute, Universidade Federal do Estado do Rio de Janeiro (UNIRIO), Rio de Janeiro, Rio de Janeiro, 20211010, Brazil; ∥ 5240 Promega Corporation, Madison, Wisconsin 53711, United States; ⊥ Scirama Psychedelic Science, São Paulo, São Paulo, 09190610, Brazil; # Usona Institute, Fitchburg, Wisconsin 53711, United States; ∇ Department of Genetics, Institute of Biology, Universidade Federal do Rio de Janeiro (UFRJ), Rio de Janeiro, Rio de Janeiro, 21941599, Brazil

**Keywords:** *N,N*-dimethyltryptamine (DMT), cell cycle, BDNF, human neural stem cells, neurosphere, induced pluripotent stem cells

## Abstract

The serotonergic psychedelic *N,N*-dimethyltryptamine (DMT) produces rapid antidepressant effects in preclinical and early clinical studies. Therapeutic benefits have been linked to sustained neural plasticity, including adult neurogenesis in rodents. Whether brief DMT exposure engages proliferative responses in human neural stem cells (NSCs) remains unresolved. Using human iPSC-derived NSCs, we found that 24 h DMT treatment increased proliferation in a concentration-dependent manner (half-maximal effect at 59.7 nM) and upregulated G1 cell-cycle regulators. DMT also shifted trophic gene expression, decreasing neurotrophin-3 while increasing nerve growth factor and brain-derived neurotrophic factor (BDNF) transcripts and intracellular BDNF protein. After washout, DMT-primed NSCs formed larger neurospheres, with progenitor and early neuronal marker composition matching controls by day 10. These findings demonstrate that brief DMT exposure is sufficient to engage proliferative and neurotrophin-associated responses in human NSCs at concentrations consistent with those reported for DMT-induced plasticity across other systems.

## Graphical abstract

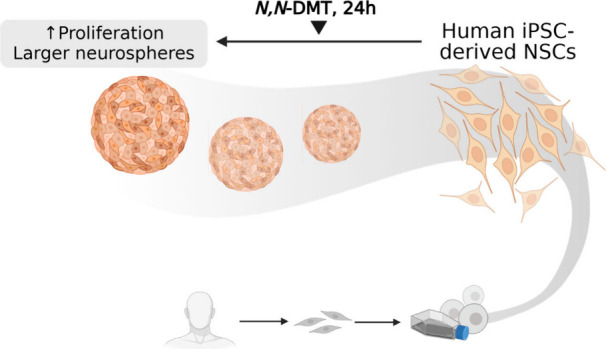

## Introduction

*N,N*-Dimethyltryptamine (DMT) is a natural psychedelic compound best known as the main psychoactive constituent of ayahuasca, a traditional Amazonian decoction typically made from *Banisteriopsis caapi* vines and *Psychotria viridis* leaves. The β-carbolines in the *B. caapi* vine act as reversible monoamine oxidase A (MAO-A) inhibitors, enabling orally ingested DMT from the *P. viridis* leaves to reach systemic circulation.^1^ In recent years, clinical and preclinical studies indicate that DMT and related psychedelics produce rapid and sustained antidepressant effects. A single dose of ayahuasca or isolated DMT has been associated with clinical improvement persisting for at least 1 week.^2–5^

The acute psychoactive effects of serotonergic psychedelics arise primarily from partial agonism at serotonin 5-HT2A receptors,^6,7^ but their therapeutic benefits for mood disorders are increasingly attributed to rapid, activity-dependent plasticity in cortical neurons. *In vitro* and *in vivo*, DMT promotes fast neurite outgrowth, spine formation and synaptogenesis at concentrations consistent with central effects estimates in humans.^8–10^ Other cellular substrates, including neurogenesis and inflammation, may contribute to DMT’s effects; however, it remains unclear if such effects produce enduring benefits for the human brain.

Neurogenesis is the multistage developmental process in which neural precursors proliferate, survive, differentiate, migrate, and ultimately integrate into pre-existing circuitry. Drug-induced adult neurogenesis has been proposed to contribute to antidepressant-like effects in rodents and nonhuman primates, but it often requires prolonged treatment, which is challenging to reconcile with the rapid, single-dose antidepressant effects reported for DMT in rodents and humans.^2,3,11–15^ Whereas chronic repeated DMT exposure was reported to reduce the NSC population in rodents, other studies found pro-neurogenic effects involving σ1R engagement, despite DMT’s relatively low affinity for this target.^16,17^ Reflecting this unresolved relationship, other serotonergic psychedelics have also produced mixed drug-, dose-, and region-dependent effects on neurogenesis in rodent models.^18–20^

A single intracerebroventricular exposure to the closely related analogue 5-*MeO*-DMT increased proliferation- and neurogenesis-related outcomes in mice,^21^ but whether acute DMT itself was similarly pro-neurogenic remained unclear. A single-dose systemic DMT study has since shown restoration of hippocampal neurogenesis markers together with reversal of anhedonia and cognitive deficits in a rodent stress-induced depression model, indicating that repeated dosing is not required for proliferation-related outcomes *in vivo*.^22^

In humans, although early studies associated chronic antidepressant treatment with increased neural stem cell (NSC) proliferation, later evidence for adult neurogenesis itself remained inconsistent, with reports ranging from lifelong persistence to near absence after childhood.^23–25^ Recent advances in tissue processing and single-cell profiling have provided new insights into persistent NSCs in the adult human brain, albeit rare and predominantly quiescent.^26–29^ These findings renew interest in fast-acting compounds with sustained effects in humans, but whether DMT engages NSCs to proliferate has not been addressed in human cells, partly due to experimental challenges.^30,31^ As a proof-of-principle, we therefore asked whether a brief DMT exposure is sufficient to induce a measurable proliferative response in human iPSC-derived NSCs, an *in vitro* stem/progenitor-cell approach suited to capture and validate human-specific cellular behaviors that are otherwise inaccessible in preclinical modeling.

## Results and Discussion

Before treatment, iPSC-derived NSCs exhibited stable expansion and neural lineage commitment (Nestin^+^, SOX2^+^, PAX6^+^, BLBP^+^) while no residual pluripotency was detected (OCT4^–^) (Supporting Information Figure S1). NSCs were further validated by the generation of neurons and astrocytes (Figure S2), consistent with prior reports using these lines (Supporting Information). DMT did not affect NSC viability across concentrations or exposure paradigms, as assessed after prolonged (72 h) or acute (4–24 h) treatment (N = 3, *p* > 0.05 for all comparisons; Figure S3A,B).

A single exposure to DMT (1 nM–10 μM) resulted in a concentration-dependent increase in the EdU^+^ cell fraction after 24 h, reaching a maximum at 1 μM, corresponding to a 23.8% increase in proliferation over vehicle (*p* = 0.0032, N = 5; Figure 1B,C). The DNA polymerase inhibitor aphidicolin (APHD; 1 μM) abolished EdU incorporation (vs vehicle and DMT 1 μM, *p* < 0.0001, N = 4), thereby confirming bona fide DNA synthesis in this assay (Figure S4A). Significant elevations persisted at 10 μM (*p* = 0.0089; Figure 1B,C). Nonlinear regression (4PL) yielded an apparent EC_50_ of ∼ 60 nM (Figure 1D). Although the present data do not resolve mechanisms, the low-nanomolar pooled potency places the proliferative response within a biologically active concentration range previously associated with DMT effects in other systems, supporting biological plausibility.^9,10,16^ ^,^ ^32,33^

**Figure 1:**
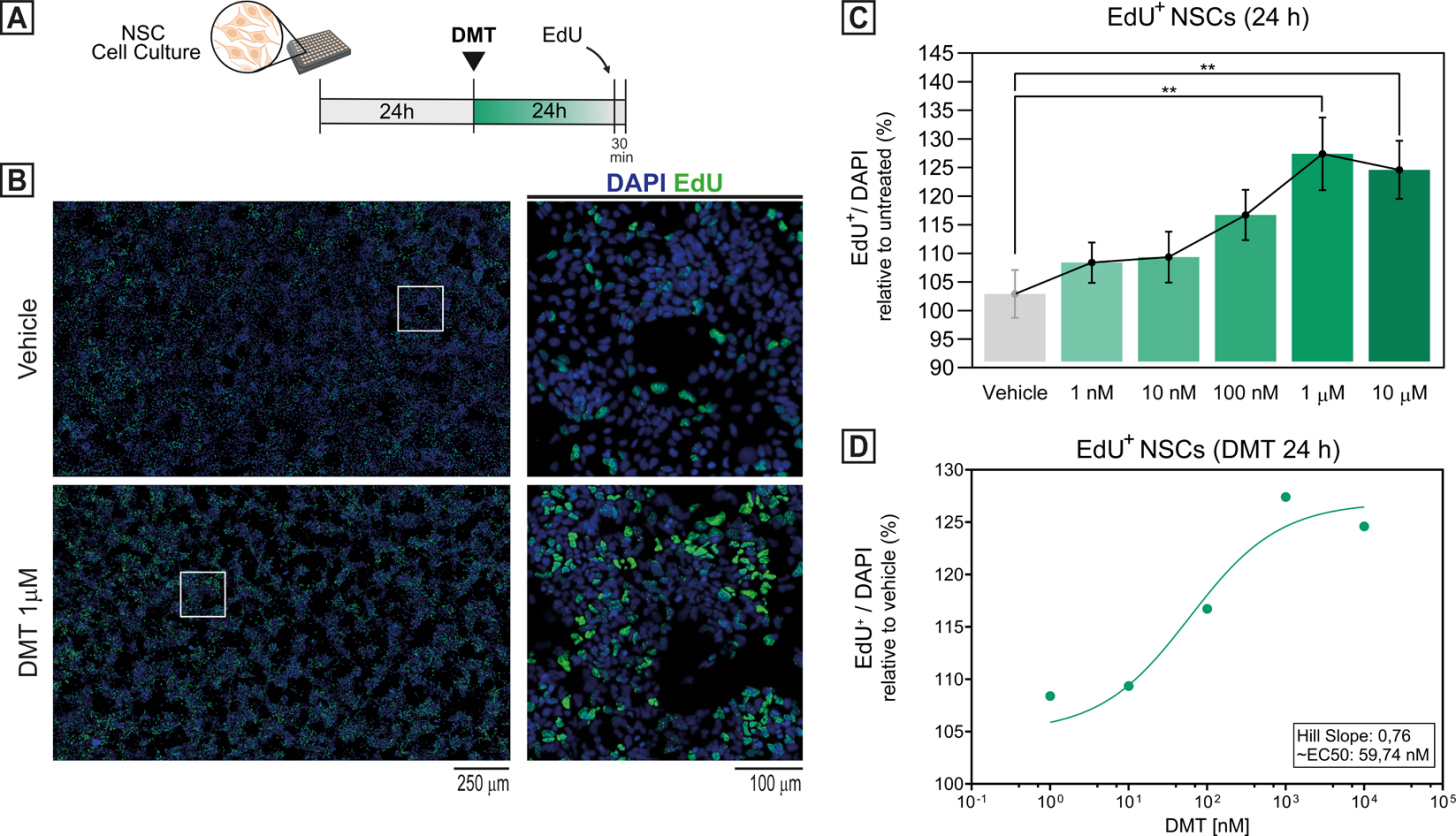
**DMT increases proliferation of human NSCs.** (A) Workflow for 24 h proliferation assays. EdU (10 μM) was added during the final 30 min of DMT exposure. (B) Representative EdU^+^ (green) and DAPI (blue) images for vehicle and DMT (1 μM) conditions. Scale bars: 250 μm (main, left) and 100 μm (insets, right). (C) EdU^+^ fraction across DMT concentrations (1 nM–10 μM), expressed as percent of untreated cells to allow inspection of potential vehicle effects (DMSO 0.04%). Symbols/bars represent experiment-level means ± SEM from *N* = 5 independent experiments using 3 iPSC-derived NSC lines (Mixed-effects model with Dunnett’s post hoc test; **p < 0.01). (D) Four-parameter logistic fit of EdU^+^ fraction relative to vehicle using the same experiment-level data set shown in C. Exact replicates per experiment and assay-to-line contribution is detailed in Table S2.

Given the near-maximal EdU response (Figure 1C), we selected DMT 1 μM to perform an initial pharmacological interrogation of receptors previously implicated in DMT-induced plasticity, including 5-HT2A, σ1R, and TrkB.^9,10,16^ Under our exploratory assay conditions, no single antagonist prevented the DMT-induced increase in EdU incorporation (Figure S4B). Co-treatment with the σ1R-antagonist BD-1063, instead, produced a small but significant increment in EdU^+^ fraction compared to DMT alone (*p* = 0.0456; Figure S4B). This finding contrasts with prior murine studies, where BD-1063 blocked DMT-induced neurosphere proliferation and neurogenesis-related outcomes *in vivo*.^16^ A second σ1R-antagonist, NE-100, also failed to prevent the DMT response but did not replicate the BD-1063-associated increase relative to DMT alone, suggesting that the modest increase observed with BD-1063 is likely antagonist-specific in our assay (Figure S4B). The pharmacological data should be interpreted as exploratory rather than mechanistic and future studies employing combinatorial and dose–response antagonism or genetic approaches are still needed to dissect the contribution of DMT receptors to proliferation in human NSCs.

In addition to EdU^+^, DMT 1 μM increased the Ki-67^+^ fraction by 66.3% at 24 h, consistent with elevated cycling (*p* = 0.0130, N = 5) (Figure 2A). G1/S cyclin *CCNE1* transcript (Cyclin E1) was reduced to 0.73-fold of vehicle (log_2_ fold change, log_2_FC = – 0.46 ± 0.09; *p* = 0.0137; N = 4; Figure 2B), whereas DMT induced an approximately 2-fold increase in the G1-phase cyclin *CCND1* transcript (Cyclin D1) (log_2_FC = 1.02 ± 0.28, *p* = 0.0341, N = 4; Figure 2B). Other G1 cyclins trended upward without reaching statistical significance. DMT increased retinoblastoma protein (pRb) phosphorylation at Ser807/811 by 40.2 ± 7.1% relative to vehicle (*p* = 0.0051, N = 3; Figure 2C), an event linked to G1 progression via Cyclin D–CDK4/6. As expected, the CDK4/6 inhibitor palbociclib (3.5 μM) reduced pRb levels to 6.7% of vehicle (−93.3 ± 2.7%; *p* = 0.0002, N = 3; Figure 2C).

**Figure 2:**
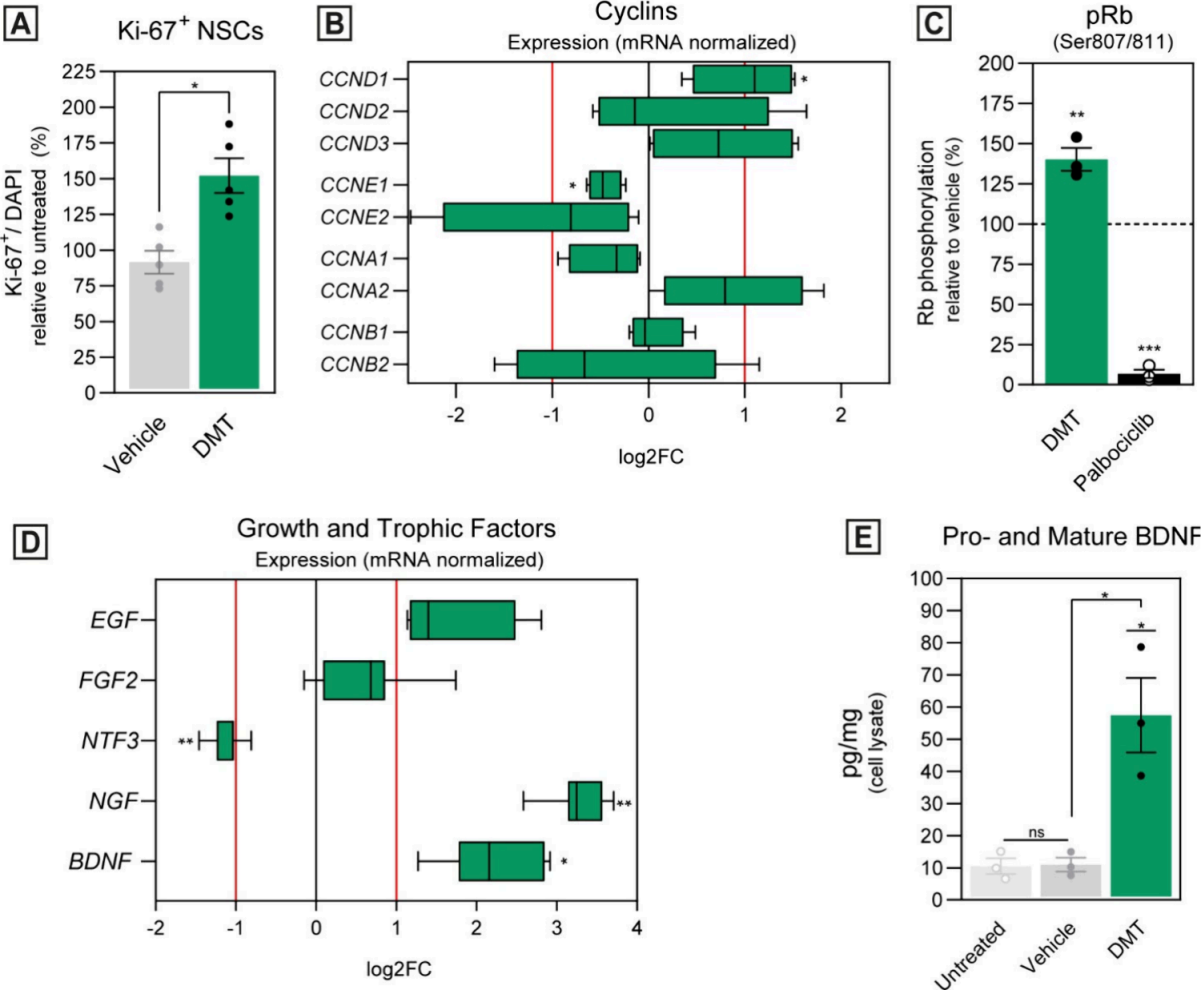
**DMT modulates G1 cell cycle regulators and trophic factor expression in human NSCs.** (A) Ki-67^+^ fraction after 24 h DMT treatment, expressed as percent of untreated cells. Symbols/bars represent experiment-level means ± SEM from *N* = 5 independent experiments using 3 iPSC-derived NSC lines (Paired *t* test, vehicle vs DMT; *p < 0.05). (B) RT-qPCR analysis of cell-cycle genes. Data are shown as boxplots of experiment-level log_2_ fold change (log_2_FC) relative to matched vehicle controls from *N* = 4 independent experiments using 2 iPSC-derived NSC lines (One-sample *t* test against 0, corresponding to no change relative to vehicle; *p < 0.05). (C) pRb (Ser807/811) levels after DMT or palbociclib (3.5 μM), expressed as percentage relative to vehicle. Symbols/bars represent experiment-level means ± SEM from *N* = 3 independent experiments using 2 iPSC-derived NSC lines (Mixed-effects model with Dunnett’s post hoc test; **p < 0.01, ***p < 0.001). (D) RT-qPCR analysis of mitogen and neurotrophin transcripts. Data are shown as boxplots of experiment-level log_2_FC relative to matched vehicle controls from *N* = 4 independent experiments using 2 iPSC-derived NSC lines (One-sample *t* test against 0; *p < 0.05, **p < 0.01). (E) Intracellular BDNF levels in NSC whole-cell lysates quantified by ELISA and normalized to total protein content. Symbols/bars represent experiment-level means ± SEM from *N* = 3 independent experiments using 2 iPSC-derived NSC lines (Mixed-effects model with Dunnett’s post hoc test; *p < 0.05). Red vertical lines in B and D indicate 2-fold down- or up-regulation relative to vehicle and statistical threshold is indicated by asterisks according to the direction of change (left for decreased, right for increased expression). Exact replicates per experiment and assay-to-line contribution is detailed in Table S2.

The concurrent *CCND1* upregulation and increase in EdU^+^, Ki-67^+^ and pRB (Ser807/811) are consistent with G1 entry, while acknowledging these are snapshot measures of cycling fractions and whether palbociclib abolishes DMT-induced EdU or pRb was not directly tested here. Future single-cell or time-resolved studies are warranted to further clarify dynamic regulatory changes underlying DMT-induced proliferation, since bulk analysis of asynchronous NSC populations lacks such resolution.

We next examined whether DMT modulated endogenous mitogen and neurotrophin transcripts in NSCs. DMT increased *EGF* (∼3-fold) and *FGF2* (∼1.5-fold) transcripts, though neither change reached significance (both *p* > 0.05 vs vehicle; N = 4, Figure 2D). The trends, nonetheless, align with progenitor expansion and cell-cycle drive.^34,35^ *NTF4* transcripts were undetectable under our conditions (data not shown), whereas *NTF3* was markedly downregulated to 0.47-fold of vehicle (log_2_FC = – 1.08 ± 0.10; *p* = 0.0099, N = 4, Figure 2D), consistent with its previously proposed role as a negative niche regulator of NSC proliferation.^36^

*NGF* and *BDNF* transcripts were upregulated by 9.5-fold (log_2_FC = 3.27 ± 0.14; *p* = 0.0018) and 4.6-fold (log_2_FC = 2.20 ± 0.39; *p* = 0.0228), respectively (N = 4, Figure 2D). Because BDNF has been more consistently implicated in psychedelic-associated plasticity, we next asked whether DMT also altered its intracellular protein levels by ELISA in this NSC model. While prior mouse studies showed only minor DMT-induced changes in BDNF,^10^ we found a marked 5.2-fold increase in total intracellular BDNF after DMT compared with vehicle (57.45 ± 11.62 pg/mg vs 10.95 ± 2.14 pg/mg; *p* = 0.0122, N = 3), consistent with mRNA upregulation (Figure 2E). These data indicate a robust BDNF protein increase in NSCs after DMT exposure, without establishing BDNF secretion, maturation state, receptor engagement, or downstream trophic signaling output. NGF protein levels and secretion were not assessed, but the nearly 10-fold increase in its transcript merits investigation in future studies of DMT-induced proliferative and plasticity-related responses.^37,38^

To assess sustained postwashout effects, NSCs were primed for 24 h with vehicle or DMT (1 μM), recovered and aggregated at identical density into single neurospheres (NPs) without further DMT exposure (Figure 3A). Following 3 days *in vitro* (DIV3) under expansion condition, media was switched to differentiation-permissive and NPs were further monitored at DIV5 and DIV10 (Figure 3A). DMT priming significantly enlarged NP projected area at DIV3 (+22.1%, *p* = 0.008) and the size trends remained positive at DIV5 and DIV10 (+12.2% and +11.6%, respectively), albeit not significant in later time points after post hoc correction (*p > 0.05*; N = 4 independent experiments; mixed-effects model, Holm-Sidak’s post hoc; Figure 3B,C). Similarly, DMT-priming effects on ATP output relative to same-plate vehicle controls were most pronounced at DIV3 by +23.8 ± 5.5% (*p* = 0.000015) followed by partial attenuation, but remaining significantly increased across DIV5 and DIV10 (+13.2 ± 3.7% and +12.6 ± 4.0%, respectively; both *p* = 0.010771; N = 7 independent experiments; mixed-effects model, Holm–Sidak’s post hoc; Figure 3D).

**Figure 3:**
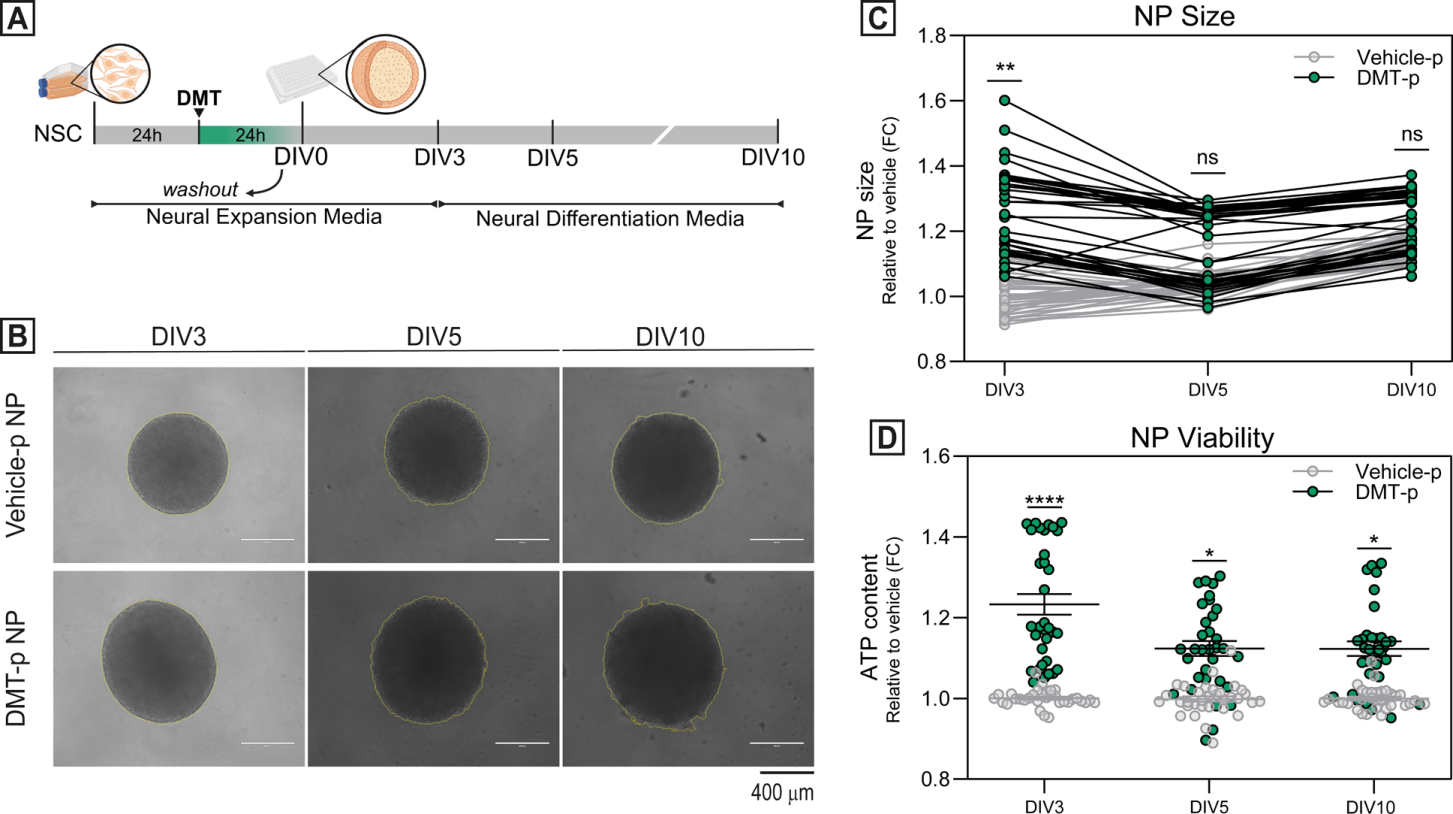
**A single DMT treatment on NSCs confers a growth advantage in neurospheres (NPs)** (A) Experimental workflow for DMT priming (1 μM, 24 h), washout, and subsequent NP formation. (B) Representative bright-field images at DIV3, DIV5, DIV10 (scale bar: 400 μm). (C) Projected area of individually tracked NPs quantified across DIV3, DIV5, and DIV10. Circles/lines represent fold change (FC) values of single NPs relative to same-plate vehicle controls across experiments (for visualization-only; vehicle: *n* = 39 NPs; DMT: *n* = 40 NPs). Means from *N* = 4 independent experiments were used for inference and confirmed consistent between 2 iPSC-derived NSC lines tested (Mixed-effects model with Holm-Sidaks’s post hoc; **p < 0.01; ns, nonsignificant). (D) ATP content measured longitudinally in tracked NPs and normalized to the mean of same-plate vehicle controls at each time point. Data are shown as FC relative to plate-matched vehicle. Circles represent FC values for individual tracked NPs across experiments (for visualization-only; vehicle: *n* = 35 NPs; DMT: *n* = 33 NPs). Results from *N* = 7 independent experiments were consistent among 3 iPSC-derived NSC lines tested and averaged for experiment-level analysis (Mixed-effects model with Holm-Sidaks’s post hoc; *p < 0.05, ****p < 0.0001). Assay-to-line contribution is detailed in Table S2.

Because larger NPs can reflect changes in growth, viability, aggregation, or cellular composition, we next asked whether the postwashout growth advantage was accompanied by gross changes in progenitor and early neuronal marker organization at DIV10.

DMT-primed and vehicle NPs showed comparable profiles by immunophenotyping of intact cryosections and dissociated-cell populations (Figure 4A–C). Nestin-positive area was similar between vehicle- and DMT-primed NPs (35.5 ± 3.7% vs 37.0 ± 5.7%, respectively; N = 4; ns, *p* > 0.05), as was βIII-tubulin (TUBB3)-positive area (48.0 ± 5.9% vs 48.1 ± 8.8%; N = 4; ns, *p* > 0.05) (Figure 4B). Among the markers quantified in intact sections, microtubule-associated protein 2 (MAP2) showed the broadest immunoreactive coverage and greater variability in both groups, consistent with its somatodendritic enrichment, but also did not differ significantly between vehicle- and DMT-primed NPs across independent experiments (56.4 ± 13.5% vs 50.8 ± 16.6%; N = 3; ns, *p* > 0.05) (Figure 4B). In dissociated NPs analyzed by flow cytometry, Nestin^+^ cells (vehicle: 97.7 ± 1.1% vs DMT: 97.3 ± 2.0%; *p* = 0.8434, N = 7) and TUBB3^+^ cells (vehicle: 96.7 ± 0.9% vs DMT: 96.4 ± 1.3%; *p* = 0.7406, N = 5) remained broadly represented in both groups, whereas MAP2 marked a smaller but similarly unchanged population (vehicle: 85.2 ± 2.8% vs DMT: 83.4 ± 3.2%; p = 0.1800, N = 7) (Figure 4C).

**Figure 4:**
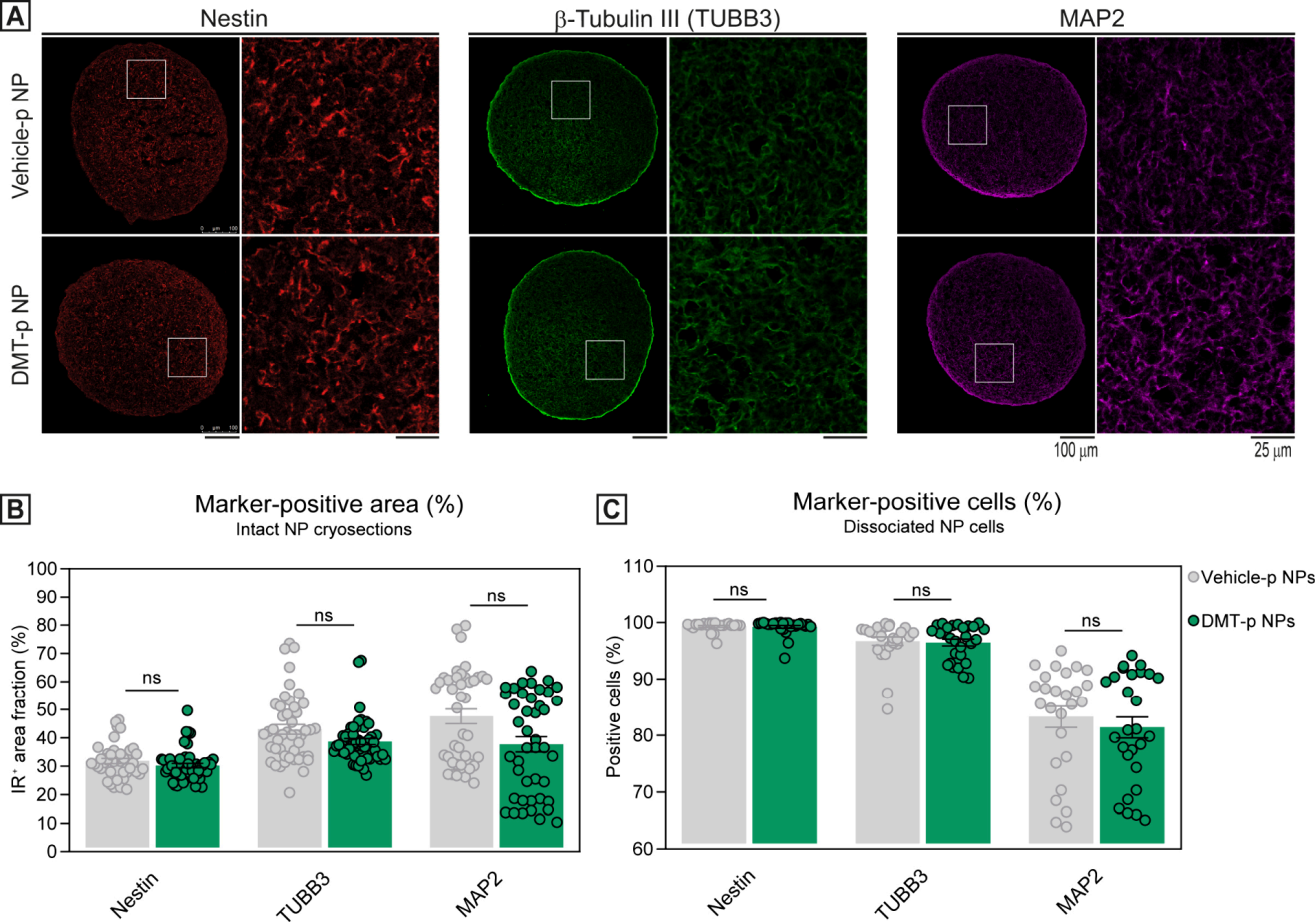
**Neurospheres (NPs) from DMT-primed NSCs showed comparable progenitor and early neuronal marker composition at DIV10.** (A) Representative immunohistochemistry for progenitor Nestin (red), immature neuronal βIII-tubulin (TUBB3; green), and early neuronal dendrite microtubule-associated protein 2 (MAP2; magenta) markers in NP cryosections at DIV10. Scale bars: 100 μm (main) and 25 μm (insets). (B) Marker-positive area was quantified in intact DIV10 NP cryosections from vehicle- and DMT-primed NSCs and consistent between NPs from 2 iPSC-derived NSC lines. Bars summarize paired experiment-level means ± SEM used for statistical inference (Nestin and TUBB3, *N* = 4; MAP2, *N* = 3; two-tailed paired *t* tests; ns, *p* > 0.05), whereas circles show individual NPs analyzed across independent experiments for transparency (Nestin IR^+^: vehicle *n* = 42, DMT *n* = 49; TUBB3 IR^+^: vehicle *n* = 47, DMT *n* = 52; MAP2 IR^+^: vehicle *n* = 42, DMT *n* = 43). (C) Marker-positive cells were quantified by flow cytometry in dissociated DIV10 NPs from 3 iPSC-derived NSC lines. Bars summarize experiment-level means used for statistical inference (Nestin^+^, *N* = 7; TUBB3^+^, *N* = 5; MAP2^+^, *N* = 7; two-tailed paired *t* tests; ns, *p* > 0.05), whereas circles show individual NP-derived measurements for transparency (Nestin^+^: vehicle *n* = 23, DMT *n* = 27; TUBB3^+^: vehicle *n* = 24, DMT *n* = 28; MAP2^+^: vehicle *n* = 26, DMT *n* = 27). Assay-to-line contribution is detailed in Table S2.

Taken together, these data identify a brief-DMT-induced proliferative state in human NSCs that carries into early 3D NP growth after drug withdrawal. No individual antagonist identified a uniquely required receptor under our exploratory assay conditions, warranting further mechanistic investigation. After washout, DMT-primed NSCs generated NPs with an early morphometric growth advantage and a smaller but persistent increase in ATP content through DIV10, without detectable gross shifts in Nestin, TUBB3, or MAP2 marker composition after transition to differentiation-permissive medium. Together with BDNF transcript upregulation and intracellular protein accumulation, these findings support further studies to determine how, or whether, these cellular changes contribute to broader plasticity-relevant mechanisms in humans.

## Methods

### Human iPS-Derived Neural Stem Cells (NSCs)

Human neural stem cells (NSCs) were generated from five independent human induced pluripotent stem cell (iPSC) lines previously reprogrammed and characterized as healthy controls (Supporting Information). Neural induction was performed as previously described.^39^ NSCs were maintained in Neural Expansion Medium (NEM) under standard culture conditions (37 °C, 5% CO_2_) and passaged every 5–7 days at approximately 80% confluence. ROCK inhibitor was used only during early expansion (≤P4). Detailed protocols are provided in the Supporting Information.

### Chemicals and Treatments

Human NSCs were treated with *N,N*-dimethyltryptamine (DMT) prepared as concentrated DMSO stocks and freshly diluted in NEM immediately before use, with final DMSO concentrations ≤ 0.04% (v/v). For validation experiments, aphidicolin (DNA polymerase α inhibitor) or palbociclib (CDK4/6 inhibitor) were coincubated with DMT. Matched vehicle controls were included in all experiments. NSCs between passages P15–P18 were seeded at 6.45 × 10^4^ cells cm^–2^ on Geltrex-coated vessels 24 h prior to treatment. Treatment allocation was randomized, and image acquisition and downstream analyses were performed blinded to condition. Detailed information on compounds and experimental procedures is provided in the Supporting Information (Table S1).

### Proliferation Assays

Proliferation was assessed after 24 h of treatment and EdU (10 μM) was added during the final 30 min of incubation. Following fixation, EdU labeling was detected by click-chemistry, and nuclei were counterstained with DAPI. Images were acquired under consistent settings using an automated imaging platform, and nuclear segmentation was performed using CellProfiler. The fraction of EdU^+^ nuclei was normalized to same-plate vehicle controls and expressed as relative percentage. Cell-cycle activity was additionally assessed by immunolabeling for Ki-67 and by quantification of retinoblastoma protein (pRb) phosphorylation at Ser807/811. pRb phosphorylation was measured using Lumit Immunoassay Cellular Systems following the manufacturer’s instructions. Each condition was analyzed in at least three technical replicates per experimental run, using at least two independent NSC lines across a minimum of three independent experiments (Table S2). Detailed protocols are provided in the Supporting Information.

### RNA Isolation and Gene Expression

Total RNA was isolated from NSCs using a silica membrane–based kit and treated with DNase I to remove genomic DNA. Complementary DNA (cDNA) was synthesized from 1 μg of total RNA using reverse transcriptase according to the manufacturer’s instructions. Quantitative PCR (qPCR) reactions were performed using either SYBR Green–based assays (*EGF* and *FGF2*) or TaqMan hydrolysis probes (*BDNF*, *NGF*, *NTF3*, *NTF4*, and cyclins) on a StepOnePlus Real-Time PCR System. Cyclin expression was assessed using a preconfigured TaqMan array for human cell-cycle genes. Relative expression was calculated using the 2^^–^ΔΔCt^ method and expressed relative to vehicle-treated controls. Cyclin expression was normalized to the mean of three validated reference genes included in the array (*18S*, *GAPDH*, *HPRT1*), whereas *EGF*, *FGF2*, and neurotrophin transcripts were normalized to *GAPDH* and *HPRT1*, all previously validated as stable in our models. For visualization, experiment-level means fold-change values were plotted as boxplots of log_2_-transformed data (log_2_FC). For qPCR, DMT-treated samples were normalized to matched vehicle controls from the same independent experiment using the 2^^–^ΔΔCt^ method, and statistical inference was performed on experiment-level log_2_FC values against 0.

### Total Intracellular BDNF Measurement

NSCs were lysed in RIPA buffer supplemented with protease inhibitors, and protein concentration was determined using a BCA assay. Total intracellular BDNF levels in whole-cell lysates were quantified using an ELISA according to the manufacturer’s protocol and normalized to total protein content. Results were expressed as pg/mg protein.

### Neurosphere (NP) Formation and Tracking

NSCs pre-exposed for 24 h to DMT (1 μM) or matched vehicle were dissociated and aggregated into single neurospheres (NPs) as previously described.^40^ Briefly, 8.0 × 10^4^ NSCs per well were seeded in ultralow attachment 96-well plates and maintained under static culture conditions. From DIV3, cultures were transitioned to differentiation medium and maintained until DIV10 with partial medium renewal every other day. Individual NPs were imaged longitudinally by bright-field microscopy at DIV3, DIV5, and DIV10, and projected area was quantified using automated image analysis with experimenter blinding. ATP content was measured in matched NPs using a luminescent assay and normalized to same-plate vehicle controls at each time point; however, per-NP cell number, DNA, or protein content were not quantified, so ATP differences could also reflect metabolic changes. Statistical inference for all results was performed on experiment-level means and the number of contributing independent experiments and iPSC-derived NSC lines are reported in the corresponding Figure legend and Supporting Information Table S2.

### Immunohistochemistry and Flow Cytometry of NPs

At DIV10, NPs were processed for immunohistochemistry or flow cytometry. For immunohistochemistry, NPs were fixed in 4% paraformaldehyde, cryoprotected, embedded in OCT, sectioned at 10 μm, and immunostained using the same protocol applied to adherent NSCs (Supporting Information). Images were acquired using confocal microscopy. For flow cytometry, NPs were dissociated into single-cell suspensions, fixed, permeabilized, and incubated with primary antibodies followed by fluorophore-conjugated secondary antibodies. Samples were analyzed on a flow cytometer, and data were processed using FlowJo software.

### Data Analysis and Statistics

Data are presented as mean ± SEM unless otherwise indicated. For all experiments, N denotes independent experimental runs. Replicates or individual neurospheres (n) were averaged within each experiment and were not treated as independent biological replicates. Statistical inference was performed on experiment-level means (N). Exact n/N values, contributing iPSC-derived NSC lines, and inferential units are provided in the figure legends and summarized in Table S2. Across the study, five independently derived human iPSC lines were used; each assay included at least 2 iPSC-derived NSC lines. Significance was defined as *p* ≤ 0.05.

## Supplementary Material



## Notes

Detailed supporting methods (pages S2–S7), NSC characterization and differentiation potential (pages S8–S9, Figures S1–S2), cell viability and assay validation experiments (pages S10–S11, Figures S3–S4), a resources and reagents table (page S12, Table S1), line-to-assay contribution (page S14, Table S2), and antibodies, primers, and probes (pages S15–S16, Tables S3–S4) (PDF)

## ○

Department of Physiological Sciences, Universidade Federal do Espírito Santo (UFES)

## Abbreviations

DMT: *N,N*-dimethyltryptamine
MAO-A: monoamine oxidase A
5-HT2A: serotonin 5-hydroxytryptamine 2A receptor
NSC: neural stem cell
σ1R: sigma-1 receptor
iPSC: induced pluripotent stem cell
SOX2: SRY-box transcription factor 2
PAX6: paired box protein 6
BLBP: brain lipid-binding protein
OCT4: octamer-binding transcription factor 4
EdU: 5-ethynyl-2′-deoxyuridine
APHD: aphidicolin
4PL: four-parameter logistic
EC50: half-maximal effective concentration
TrkB: tropomyosin receptor kinase B
EGF: epidermal growth factor
FGF2: fibroblast growth factor 2
NTF3: neurotrophin 3
NTF4: neurotrophin 4
BDNF: brain-derived neurotrophic factor
NGF: nerve growth factor
pRb: phosphorylated retinoblastoma protein
CDK: cyclin-dependent kinase
NP: neurosphere
DIV: days in vitro
log_2_FC: log2 fold change
SEM: standard error of the mean

## References

[1] Aicher, H. D. ; Mueller, M. J. ; Dornbierer, D. A. ; Suay, D. ; Elsner, C. ; Wicki, I. ; Meling, D. ; Caflisch, L. ; Hempe, A. ; Steinhart, C. ; Mueller, J. ; Von Rotz, R. ; Kleim, B. ; Scheidegger, M. Potential Therapeutic Effects of an Ayahuasca-Inspired N,N-DMT and Harmine Formulation: A Controlled Trial in Healthy Subjects. Front. Psychiatry 2024, 14. 10.3389/fpsyt.2023.1302559. PMC10804806 38264636

[2] Falchi-Carvalho M., Barros H., Bolcont R., Laborde S., Wießner I., Ruschi B Silva S., Montanini D., Barbosa D. C., Teixeira E., Florence-Vilela R., Almeida R., de Macedo R. K. A., Arichelle F., Pantrigo É. J., Costa-Macedo J. V., Arcoverde E., Galvão-Coelho N., Araujo D. B., Palhano-Fontes F. (2025). The Antidepressant
Effects of Vaporized *N*, *N* -Dimethyltryptamine:
An Open-Label Pilot Trial
in Treatment-Resistant Depression. Psychedelic
Medicine.

[3] Palhano-Fontes F., Barreto D., Onias H., Andrade K. C., Novaes M. M., Pessoa J. A., Mota-Rolim S. A., Osório F. L., Sanches R., Dos Santos R. G., Tófoli L. F., De Oliveira Silveira G., Yonamine M., Riba J., Santos F. R., Silva-Junior A. A., Alchieri J. C., Galvão-Coelho N. L., Lobão-Soares B., Hallak J. E. C., Arcoverde E., Maia-De-Oliveira J. P., Araújo D. B. (2019). Rapid Antidepressant Effects of the
Psychedelic Ayahuasca in Treatment-Resistant Depression: A Randomized
Placebo-Controlled Trial. Psychol. Med..

[4] Timmermann C., Zeifman R. J., Erritzoe D., Nutt D. J., Carhart-Harris R. L. (2024). Effects
of DMT on Mental Health Outcomes in Healthy Volunteers. Sci. Rep..

[5] Luan L. X., Eckernäs E., Ashton M., Rosas F. E., Uthaug M. V., Bartha A., Jagger S., Gascon-Perai K., Gomes L., Nutt D. J., Erritzøe D., Carhart-Harris R. L., Timmermann C. (2024). Psychological and Physiological Effects
of Extended DMT. Journal of Psychopharmacology.

[6] Valle M., Maqueda A. E., Rabella M., Rodríguez-Pujadas A., Antonijoan R. M., Romero S., Alonso J. F., Mañanas M. À., Barker S., Friedlander P., Feilding A., Riba J. (2016). Inhibition
of Alpha Oscillations through Serotonin-2A Receptor Activation Underlies
the Visual Effects of Ayahuasca in Humans. European
Neuropsychopharmacology.

[7] Halberstadt A. L., Chatha M., Klein A. K., Wallach J., Brandt S. D. (2020). Correlation
between the Potency of Hallucinogens in the Mouse Head-Twitch Response
Assay and Their Behavioral and Subjective Effects in Other Species. Neuropharmacology.

[8] Eckernäs E., Timmermann C., Carhart-Harris R., Röshammar D., Ashton M. (2022). Population Pharmacokinetic/Pharmacodynamic Modeling
of the Psychedelic Experience Induced by N,N-Dimethyltryptamine -
Implications for Dose Considerations. Clin.
Transl. Sci..

[9] Vargas M. V., Dunlap L. E., Dong C., Carter S. J., Tombari R. J., Jami S. A., Cameron L. P., Patel S. D., Hennessey J. J., Saeger H. N., McCorvy J. D., Gray J. A., Tian L., Olson D. E. (2023). Psychedelics Promote Neuroplasticity through the Activation
of Intracellular 5-HT2A Receptors. Science.

[10] Ly C., Greb A. C., Cameron L. P., Wong J. M., Barragan E. V., Wilson P. C., Burbach K. F., Soltanzadeh Zarandi S., Sood A., Paddy M. R., Duim W. C., Dennis M. Y., McAllister A. K., Ori-McKenney K. M., Gray J. A., Olson D. E. (2018). Psychedelics
Promote Structural and Functional Neural Plasticity. Cell Rep..

[11] de
Sousa-Silva A. N., Moura C. de A., de Oliveira Torres C. I., Marques V. B., do Nascimento Silva J.
M., Lobão-Soares B., Ruschi Silva S., Galvão-Coelho N. L., Palhano-Fontes F., Araújo D. B. de, da Silva E. D., Gavioli E. C. (2026). N,N-Dimethyltryptamine
Elicits Antidepressant and Anxiolytic Effects in Helpless Mice: A
Comparative Study with S-Ketamine. Neuropharmacology.

[12] Malberg J. E., Hen R., Madsen T. M. (2021). Adult Neurogenesis
and Antidepressant Treatment: The
Surprise Finding by Ron Duman and the Field 20 Years Later. Biol. Psychiatry.

[13] Ohira K., Hagihara H., Miwa M., Nakamura K., Miyakawa T. (2019). Fluoxetine-Induced
Dematuration of Hippocampal Neurons and Adult Cortical Neurogenesis
in the Common Marmoset. Mol. Brain.

[14] Wu M. V., Shamy J. L., Bedi G., Choi C.-W. J., Wall M. M., Arango V., Boldrini M., Foltin R. W., Hen R. (2014). Impact of
Social Status and Antidepressant Treatment on Neurogenesis in the
Baboon Hippocampus. Neuropsychopharmacology.

[15] Perera T. D., Dwork A. J., Keegan K. A., Thirumangalakudi L., Lipira C. M., Joyce N., Lange C., Higley J. D., Rosoklija G., Hen R., Sackeim H. A., Coplan J. D. (2011). Necessity
of Hippocampal Neurogenesis for the Therapeutic Action of Antidepressants
in Adult Nonhuman Primates. PLoS One.

[16] Morales-Garcia J. A., Calleja-Conde J., Lopez-Moreno J. A., Alonso-Gil S., Sanz-SanCristobal M., Riba J., Perez-Castillo A. (2020). N,N-Dimethyltryptamine
Compound Found in the Hallucinogenic Tea Ayahuasca, Regulates Adult
Neurogenesis in Vitro and in Vivo. Translational
Psychiatry 2020 10:1.

[17] Borbély E., Varga V., Szögi T., Schuster I., Bozsó Z., Penke B., Fülöp L. (2022). Impact of
Two Neuronal Sigma-1 Receptor
Modulators, PRE084 and DMT, on Neurogenesis and Neuroinflammation
in an Aβ1–42-Injected, Wild-Type Mouse Model of AD. Int. J. Mol. Sci..

[18] Banasr M., Hery M., Printemps R., Daszuta A. (2004). Serotonin-Induced Increases
in Adult Cell Proliferation and Neurogenesis Are Mediated through
Different and Common 5-HT Receptor Subtypes in the Dentate Gyrus and
the Subventricular Zone. Neuropsychopharmacology.

[19] Catlow B. J., Song S., Paredes D. A., Kirstein C. L., Sanchez-Ramos J. (2013). Effects of
Psilocybin on Hippocampal Neurogenesis and Extinction of Trace Fear
Conditioning. Exp. Brain Res..

[20] Jha S., Rajendran R., Fernandes K. A., Vaidya V. A. (2008). 5-HT2A/2C Receptor
Blockade Regulates Progenitor Cell Proliferation in the Adult Rat
Hippocampus. Neurosci. Lett..

[21] Lima da Cruz, R. V. ; Moulin, T. C. ; Petiz, L. L. ; Leão, R. N. A Single Dose of 5-MeO-DMT Stimulates Cell Proliferation, Neuronal Survivability, Morphological and Functional Changes in Adult Mice Ventral Dentate Gyrus. Front. Mol. Neurosci. 2018, 11. 10.3389/fnmol.2018.00312. PMC6131656 30233313

[22] Lima
da Cruz R. V., Costa R. B. G. de M., de Queiroz G. M., Stojanovic T., Moulin T. C., Leão R. N. (2026). Single-Dose
DMT Reverses Anhedonia and Cognitive Deficits via Restoration of Neurogenesis
in a Stress-Induced Depression Model. Transl.
Psychiatry.

[23] Sorrells S. F., Paredes M. F., Cebrian-Silla A., Sandoval K., Qi D., Kelley K. W., James D., Mayer S., Chang J., Auguste K. I., Chang E. F., Gutierrez A. J., Kriegstein A. R., Mathern G. W., Oldham M. C., Huang E. J., Garcia-Verdugo J. M., Yang Z., Alvarez-Buylla A. (2018). Human Hippocampal
Neurogenesis Drops Sharply in Children to Undetectable Levels in Adults. Nature.

[24] Moreno-Jiménez E. P., Flor-García M., Terreros-Roncal J., Rábano A., Cafini F., Pallas-Bazarra N., Ávila J., Llorens-Martín M. (2019). Adult Hippocampal Neurogenesis
Is
Abundant in Neurologically Healthy Subjects and Drops Sharply in Patients
with Alzheimer’s Disease. Nat. Med..

[25] Boldrini M., Underwood M. D., Hen R., Rosoklija G. B., Dwork A. J., John Mann J., Arango V. (2009). Antidepressants Increase
Neural Progenitor Cells in the Human Hippocampus. Neuropsychopharmacology.

[26] Flor-García M., Terreros-Roncal J., Moreno-Jiménez E. P., Ávila J., Rábano A., Llorens-Martín M. (2020). Unraveling Human Adult
Hippocampal Neurogenesis. Nature Protocols 2020
15:2.

[27] Baig S., Nadaf J., Allache R., Le P. U., Luo M., Djedid A., Nkili-Meyong A., Safisamghabadi M., Prat A., Antel J., Guiot M.-C., Petrecca K. (2024). Identity and
Nature of Neural Stem Cells in the Adult Human Subventricular Zone. iScience.

[28] Dumitru I., Paterlini M., Zamboni M., Ziegenhain C., Giatrellis S., Saghaleyni R., Björklund Å., Alkass K., Tata M., Druid H., Sandberg R., Frisén J. (2025). Identification of Proliferating Neural Progenitors
in the Adult Human Hippocampus. Science (1979)..

[29] Donega V., van der Geest A. T., Sluijs J. A., van Dijk R. E., Wang C. C., Basak O., Pasterkamp R. J., Hol E. M. (2022). Single-Cell Profiling
of Human Subventricular Zone Progenitors Identifies SFRP1 as a Target
to Re-Activate Progenitors. Nat. Commun..

[30] Chaves C., Dos Santos R. G., Dursun S. M., Tusconi M., Carta M. G., Brietzke E., Hallak J. E. C. (2024). Why N,N-Dimethyltryptamine Matters:
Unique Features and Therapeutic Potential beyond Classical Psychedelics. Front. Psychiatry.

[31] Lima
da Cruz R. V., Leão R. N., Moulin T. C. (2024). Effects of Psychedelics
on Neurogenesis and Broader Neuroplasticity: A Systematic Review. Mol. Med..

[32] László, M. J. ; Vigh, J. P. ; Kocsis, A. E. ; Porkoláb, G. ; Hoyk, Z. ; Polgár, T. ; Walter, F. R. ; Szabó, A. ; Djurovic, S. ; Merkely, B. ; Alpár, A. ; Frecska, E. ; Nagy, Z. ; Deli, M. A. ; Nardai, S. *N*,*N*-Dimethyltryptamine Mitigates Experimental Stroke by Stabilizing the Blood-Brain Barrier and Reducing Neuroinflammation. Sci. Adv. 2025, 11 (33). 10.1126/sciadv.adx5958. PMC12346280 40802766

[33] Szabo A., Kovacs A., Riba J., Djurovic S., Rajnavolgyi E., Frecska E. (2016). The Endogenous Hallucinogen
and Trace Amine N,N-Dimethyltryptamine
(DMT) Displays Potent Protective Effects against Hypoxia via Sigma-1
Receptor Activation in Human Primary IPSC-Derived Cortical Neurons
and Microglia-Like Immune Cells. Front. Neurosci..

[34] Kang W., Hebert J. M. (2015). FGF Signaling Is
Necessary for Neurogenesis in Young
Mice and Sufficient to Reverse Its Decline in Old Mice. J. Neurosci..

[35] Zhang Q., Liu G., Wu Y., Sha H., Zhang P., Jia J. (2011). BDNF Promotes
EGF-Induced Proliferation and Migration of Human Fetal Neural Stem/Progenitor
Cells via the PI3K/Akt Pathway. Molecules.

[36] Delgado A. C., Ferrón S. R., Vicente D., Porlan E., Perez-Villalba A., Trujillo C. M., D'Ocón P., Fariñas I. (2014). Endothelial
NT-3 Delivered by Vasculature and CSF Promotes Quiescence of Subependymal
Neural Stem Cells through Nitric Oxide Induction. Neuron.

[37] Scardigli R., Capelli P., Vignone D., Brandi R., Ceci M., Regina F., Piras E., Cintoli S., Berardi N., Capsoni S., Cattaneo A. (2014). Neutralization of Nerve
Growth Factor
Impairs Proliferation and Differentiation of Adult Neural Progenitors
in the Subventricular Zone. Stem Cells.

[38] Colitti, N. ; Desmoulin, F. ; Le Friec, A. ; Labriji, W. ; Robert, L. ; Michaux, A. ; Conchou, F. ; Cirillo, C. ; Loubinoux, I. Long-Term Intranasal Nerve Growth Factor Treatment Favors Neuron Formation in de Novo Brain Tissue. Front. Cell. Neurosci. 2022, 16. 10.3389/fncel.2022.871532. PMC9345199 35928573

[39] Yan Y., Shin S., Jha B. S., Liu Q., Sheng J., Li F., Zhan M., Davis J., Bharti K., Zeng X., Rao M., Malik N., Vemuri M. C. (2013). Efficient and Rapid Derivation of
Primitive Neural Stem Cells and Generation of Brain Subtype Neurons
From Human Pluripotent Stem Cells. Stem Cells
Transl. Med..

[40] Pedrosa C. da S. G., Goto-Silva L., Temerozo J. R., Souza L. R. Q., Vitória G., Ornelas I. M., Karmirian K., Mendes M. A., Gomes I. C., Sacramento C. Q., Fintelman-Rodrigues N., Cardoso Soares V., Silva Gomes Dias S. da, Salerno J. A., Puig-Pijuan T., Oliveira J. T., Aragão L. G. H.
S., Torquato T. C. Q., Veríssimo C., Biagi D., Cruvinel E. M., Dariolli R., Furtado D. R., Borges H. L., Bozza P. T., Rehen S., Moreno L Souza T., Guimarães M. Z. P. (2021). Non-Permissive
SARS-CoV-2 Infection in Human Neurospheres. Stem Cell Res..

