# Supplementary material for: Proliferative Effects of the Psychedelic N,N-Dimethyltryptamine (DMT) in Human Neural Stem Cells
Source: ACS Chem Neurosci. 2026 Jul 10;17(15):2901–9. doi: 10.1021/acschemneuro.6c00209 (PMC13449763; doi:10.1021/acschemneuro.6c00209)
Supplement: Supplementary file 1 [file cn6c00209_si_001.pdf]

Proliferative effects of the psychedelic *N,N*-Dimethyltryptamine (DMT) in human neural stem cells

José Alexandre Salerno[1,2,3], Elizabeth R. Dominguez[4], Karina Karmirian[1,5], Breno A.B.M.S. Arrais[1,3], Juliano Alves[4], Giovanna Erjautz[1,3], Kennedy Kroening[4], Leticia R. Q. Souza[1], Isis Ornelas[1], Stevens Rehen[1,4,6,7,*]

[1] D'Or Institute for Research and Education (IDOR), Rio de Janeiro, Rio de Janeiro, BR 22281100
[2] Graduate Program in Morphological Sciences, Institute of Biomedical Sciences, Universidade Federal do Rio de Janeiro (UFRJ), Rio de Janeiro, Rio de Janeiro, BR 21941599
[3]Department of Morphological Sciences, Biomedical Institute, Universidade Federal do Estado do Rio de Janeiro (UNIRIO), Rio de Janeiro, Rio de Janeiro, BR 20211010
[4]Promega Corporation, Madison, WI, US 53711
[5]Scirama Psychedelic Science, São Paulo, São Paulo, BR 09190610
[6]Usona Institute, Fitchburg, WI, US 53711
[7]Department of Genetics, Institute of Biology, Universidade Federal do Rio de Janeiro (UFRJ), Rio de Janeiro, Rio de Janeiro, BR 21941599
[*]Corresponding author: stevens.rehen@idor.org

Supporting information (SI)

Table of contents

Supporting methods..**S2**

Supplementary figures..**S8**

Figure S1..S8

Figure S2..S9

Figure S3..S10

Figure S4..S11

Supplementary tables..**S12**

Table S1..S12

Table S2..S14

Table S3..S15

Table S4..S16

References..**S17**

Supporting methods

Human iPSC-Derived Neural Stem Cells (NSCs)

The derivation and experimental use of all human cell lines were conducted in accordance with institutional and national ethical guidelines. Human iPSCs were purchased from the Coriell Institute for Medical Research repository (GM23279A) or produced at D'Or Institute for Research and Education (IDOR) with CytoTune™-iPS 2.0 Sendai Reprogramming Kit (Invitrogen) from skin fibroblasts, urine epithelial cells or endothelial cells as previously described and characterized.[1–6] Detailed resource information is provided in Supplementary Table S1.

Human iPSCs were differentiated into NSCs using PSC Neural Induction Medium (Thermo Fisher Scientific) following the dual-SMAD inhibition protocol described.[7] After 7 days of neural induction, cells were dissociated using Accutase and replated onto Geltrex-coated cultureware in Neural Expansion Medium (NEM). NEM consisted of Neurobasal Medium and Advanced DMEM/F-12 (1:1) supplemented with 2% Neural Induction Supplement (NIS) (Supplementary Table S1). Cultures were maintained at 37 °C in a humidified atmosphere with 5% CO_2. Medium was refreshed every 48 h, and cells were passaged every 5–7 days at approximately 80% confluence. ROCK inhibitor Y-27632 (5 µM) was included only during early expansion (passages ≤P4). Mycoplasma contamination was assessed by MycoAlert™ and confirmed negative within four weeks before use.

Immunocytochemistry (NSC)

NSCs were fixed with 4% paraformaldehyde for 15 min at room temperature (RT), washed three times with PBS, and permeabilized with PBS containing 0.3% Triton X-100 for 15 min. Cells were then blocked in PBS containing 3% bovine serum albumin for 2 h at RT. Primary antibodies were diluted in blocking solution and incubated overnight at 4 °C. After three PBS washes, Alexa Fluor–conjugated secondary antibodies (Supplementary Table S3) were applied for 1.5 h RT. Nuclei were counterstained with DAPI (300 nM, 10 min). Images were acquired using a Leica TCS SP8 confocal microscope equipped with a 63× oil-immersion objective, maintaining identical acquisition settings across conditions. Antibodies, suppliers, and working dilutions are listed in Supplementary Table S3.

Neuronal differentiation assay

NSCs (2×10^6 cells) were electroporated with pT2-GFP (3 µg) and SB100X transposase (1 µg) using a Nucleofector II system (Lonza). Cells were recovered in NEM supplemented with Y-27632 (5 µM) and seeded onto 60 mm Geltrex-coated dishes until reaching approximately 80% confluence. Cells were then replated at 2×10^6 cells per 60 mm dish onto poly-L-ornithine–coated (100 µg mL^{-1}) and laminin-coated (10 µg mL^{-1}) cultureware for neuronal differentiation as previously described.[4,5] After 48 h, the medium was replaced with differentiation medium composed of Neurobasal and DMEM/F-12 (1:1) supplemented with B27 (1×) and N2 (1×). Cultures were maintained for 40 days with medium replacement every 4 days. Cells were subsequently replated at 1.3×10^5 cells cm^{-2} onto final coated glass vessels and maintained for an additional 10 days (total DIV 51) prior to fixation and immunolabeling for synaptophysin. Reagents, antibodies and working dilutions are detailed in Supplementary Tables S1 and S3.

Astrocyte differentiation assay

Astrocytes were derived from human NSCs following a previously established protocol.[2,3] Briefly, NSCs were seeded onto Geltrex-coated flasks at a density of 1.25×10^6 cells per T-25 flask. On the following day, NEM was replaced with Astrocyte Induction Medium (AIM) composed of DMEM/F-12 supplemented with N2 (1×) and 1% heat-inactivated fetal bovine serum. AIM was renewed every 2 days for 21 days. At approximately 90% confluence, cultures were dissociated with Accutase and passed at a 1:4 ratio onto freshly coated 0.5× Geltrex culture ware. After 21 days of induction, AIM was replaced with Astrocyte Maturation Medium (DMEM/F-12 supplemented with 10% fetal bovine serum), and cultures were maintained for at least 15 weeks with twice-weekly medium changes and passages every two weeks. Mature astrocytes exhibited polygonal to stellate morphology and were identified by immunolabeling. Reagents, antibodies and working dilutions are listed in Supplementary Tables S1 and S3.

Cell viability assays (2D)

Cell viability was assessed after 4 and 24 h of DMT (1 µM) exposure using a fluorometric assay based on cleavage of the glycyl-phenylalanyl-amino-fluorocoumarin (GF-AFC) substrate. The CellTiter-Fluor™ reagent was prepared according to the manufacturer's instructions and added directly to the cultures. After 30 min of incubation at 37 °C,

fluorescence was measured using a GloMax® Explorer plate reader (excitation 380–400 nm; emission 505 nm). Terminal viability was evaluated after 72 h of exposure using a colorimetric assay based on reduction of the tetrazolium salt MTT (0.5 mg mL^{-1}, 3 h, 37 °C, 5% CO_2). Formazan crystals were solubilized in DMSO (100 µL per well), and absorbance was measured at 560 nm with background correction at 690 nm using an Infinite® 200 PRO plate reader. Values were normalized to same-plate controls. Each condition included at least six technical replicates per independent experiment.

EdU Proliferation Assay

NSCs were seeded onto Geltrex-coated 96-well plates and allowed to recover for 24 h prior to treatment with vehicle, DMT, or aphidicolin (DNA polymerase α inhibitor, 1 µM). Where indicated, cultures were pre-incubated for 1 h before DMT with pharmacological antagonists or inhibitors, including ketanserin ($5\text{-}HT_{2}A$ antagonist, 100 nM), BD-1063 (σ_1R antagonist, 1 µM), NE-100 (σ_1R antagonist, 1 µM) or ANA-12 (TrkB antagonist, 10 µM). Antagonists were maintained throughout DMT exposure, and matched vehicle controls were included in all experiments (please refer to Supplementary Table S1 for reagent details). EdU (10 µM) was added during the final 30 min of treatment. Cells were then fixed, and EdU incorporation was detected using the Click-iT™ EdU imaging kit. Nuclei were counterstained with DAPI (300 nM, 15 min, RT). Images were acquired using a Cytation™ 5 automated imager (Biotek) under identical acquisition settings and analyzed with blinded pipelines in CellProfiler v4.2. The fraction of EdU^+ nuclei was normalized to same-plate controls and expressed as relative percentage.

pRb luminescence assay

Phosphorylation of retinoblastoma protein (pRb) at Ser807/811 was quantified using a Lumit® Immunoassay Cellular Systems assay (Promega). NSCs were seeded in 96-well plates at a density of 6.45×10^4 cells cm^{-2}, allowed to recover for 24 h, and treated for an additional 24 h with DMT, palbociclib, or vehicle. Cells were lysed in buffer containing 0.02% digitonin supplemented with protease and phosphatase inhibitors, with gentle agitation (800 rpm) for 40 min at room temperature. Lysates were incubated sequentially with provided antibodies recognizing phospho-Rb (Ser807/811) and total Rb, followed by species-specific SmBiT- and LgBiT-conjugated secondary antibodies (Table S3).

Luminescence was measured on a plate reader, background-subtracted, and normalized to vehicle-treated controls.

Quantitative PCR assays

SYBR Green–based quantitative PCR (qPCR) was used to quantify *EGF* and *FGF2* transcripts using custom forward and reverse primers (200 nM each) (Supplementary Table S4). Reactions were prepared with GoTaq® qPCR Master Mix containing BRYT Green® and a passive reference dye, using 1 ng μL^{-1} cDNA per reaction. Thermal cycling conditions consisted of an initial denaturation at 95 °C for 3 min, followed by 40 cycles of 95 °C for 15 s, 60 °C for 15 s, and 72 °C for 15 s. Melt-curve analysis was performed to confirm product specificity. Neurotrophin transcripts (*BDNF*, *NGF*, *NTF3*, and *NTF4*) were quantified using TaqMan® hydrolysis probes (Supplementary Table S4) in reactions with GoTaq® Probe qPCR Master Mix. Thermal cycling consisted of polymerase activation at 95 °C for 2 min, followed by 40 cycles of 95 °C for 15 s and 60 °C for 60 s. All reactions were performed in 96-well optical plates with a final reaction volume of 10 µL, run in technical triplicates, with no-template controls included. Cyclin expression was assessed using a preconfigured TaqMan™ Array Human Cyclins & Cell Cycle Regulation panel with TaqMan™ Fast Advanced Master Mix, following the manufacturer's recommended cycling conditions.

For qPCR analyses, technical replicate Cq values were first averaged within each sample per experiment. For each independent experiment, ΔΔCt values were calculated by normalizing DMT-treated samples to the matched vehicle condition from the same experimental run. Fold-change values were $\log_2$-transformed, and statistical inference was performed on experiment-level $\log_2$FC values against zero, corresponding to no change relative to vehicle.

BDNF protein measurement

Cells were lysed in 100 µL of RIPA buffer containing protease inhibitors. Lysates were homogenized, centrifuged at 14,000 × g for 15 min, and clarified supernatants were collected. Total protein concentration was determined using a BCA assay with bovine serum albumin standards. Total BDNF levels were quantified using a Human Total BDNF Quantikine® ELISA according to the manufacturer's instructions, with samples diluted 1:2 prior to analysis. Absorbance was measured at 450 nm with background

correction at 560 nm using a GloMax® Discover plate reader. BDNF concentrations were normalized to total protein content and expressed as pg BDNF per mg total protein.

Neurosphere (NP) formation

NSCs pre-exposed for 24 h to DMT (1 µM; 0.004% DMSO) or vehicle were dissociated with Accutase and resuspended in NEM. Single neurospheres were generated as previously described.[8] Briefly, NSCs were seeded into ultra-low attachment 96-well plates at a density of 8.0×10^4 cells per well in a final volume of 200 µL, centrifuged at 300 × g for 3 min, and maintained at 37 °C in a humidified atmosphere with 5% CO_2. On day in vitro 3 (DIV3), the medium was replaced with differentiation medium composed of Neurobasal and DMEM/F-12 (1:1) supplemented with B27 and N2. Partial medium changes (180 µL) were performed on DIV5 and DIV7. Neurospheres were maintained until DIV10. Neurospheres randomly chosen at DIV3 were imaged by bright-field microscopy using an EVOS™ XL Core (Thermo Fisher Scientific). Images were analyzed using blinded pipelines in CellProfiler v4.2.

Cell viability assays (3D)

Cell viability in neurospheres was assessed by ATP quantification using the CellTiter-Glo® 3D Cell Viability Assay. Individual neurospheres were transferred to white-walled 96-well plates containing 100 µL of culture medium. An equal volume (100 µL) of CellTiter-Glo® 3D reagent was added to each well, followed by incubation for 30 min at room temperature on a rotating shaker (600 rpm). Luminescence was measured using a GloMax® Discover plate reader and normalized to same-plate vehicle controls.

Immunohistochemistry (IHC) of DIV10 Neurospheres

At DIV10, neurospheres were collected and fixed in 4% paraformaldehyde prepared in conditioned medium for 1 h at RT. Samples were washed with PBS, cryoprotected in 30% (w/v) sucrose for 1 h, embedded in OCT compound (Taxon), and frozen at −80 °C for at least 24 h. Blocks were sectioned at 10 µm using a cryostat (Leica CM1860) and mounted onto microscopy slides. For immunostaining, sections were equilibrated at 37 °C for 30 min and washed three times with PBS to remove residual OCT. Immunohistochemistry was then performed using the same protocol described for NSC immunocytochemistry and images of one section per neurosphere were acquired using a Leica TCS SP8 confocal microscope with a 20× oil-immersion objective, maintaining identical laser power and

acquisition settings across conditions. Antibodies and working dilutions are listed in Supplementary Table S3.

Flow cytometry of DIV10 Neurospheres

Neurospheres cultured in 96-well plates were washed once with calcium- and magnesium-free DPBS and dissociated into single-cell suspensions by incubation with Accutase (100 µL per well) for 30 min at 37 °C. Gentle trituration was applied to facilitate dissociation, and the reaction was quenched with an equal volume of DPBS. Cells were transferred to V-bottom 96-well plates, pelleted by centrifugation at 500 × g for 5 min, resuspended in fixation/permeabilization solution (BD), and incubated for 20 min at 4 °C. Following fixation, cells were washed twice with Perm / Wash (BD) solution and incubated overnight at 4 °C with primary antibodies diluted in permeabilization buffer. Antibodies and working dilutions are provided in Supplementary Table S3. After washing, cells were incubated for 1 h at 4 °C in the dark with fluorophore-conjugated secondary antibodies. After two additional washes, cells were resuspended in FACS buffer (1% FBS in DPBS), filtered through 40 µm cell strainer caps, and analyzed on a BD LSRFortessa™ or BD Accuri C6 Plus flow cytometer. Data were processed using FlowJo™ software.

Supplementary figures

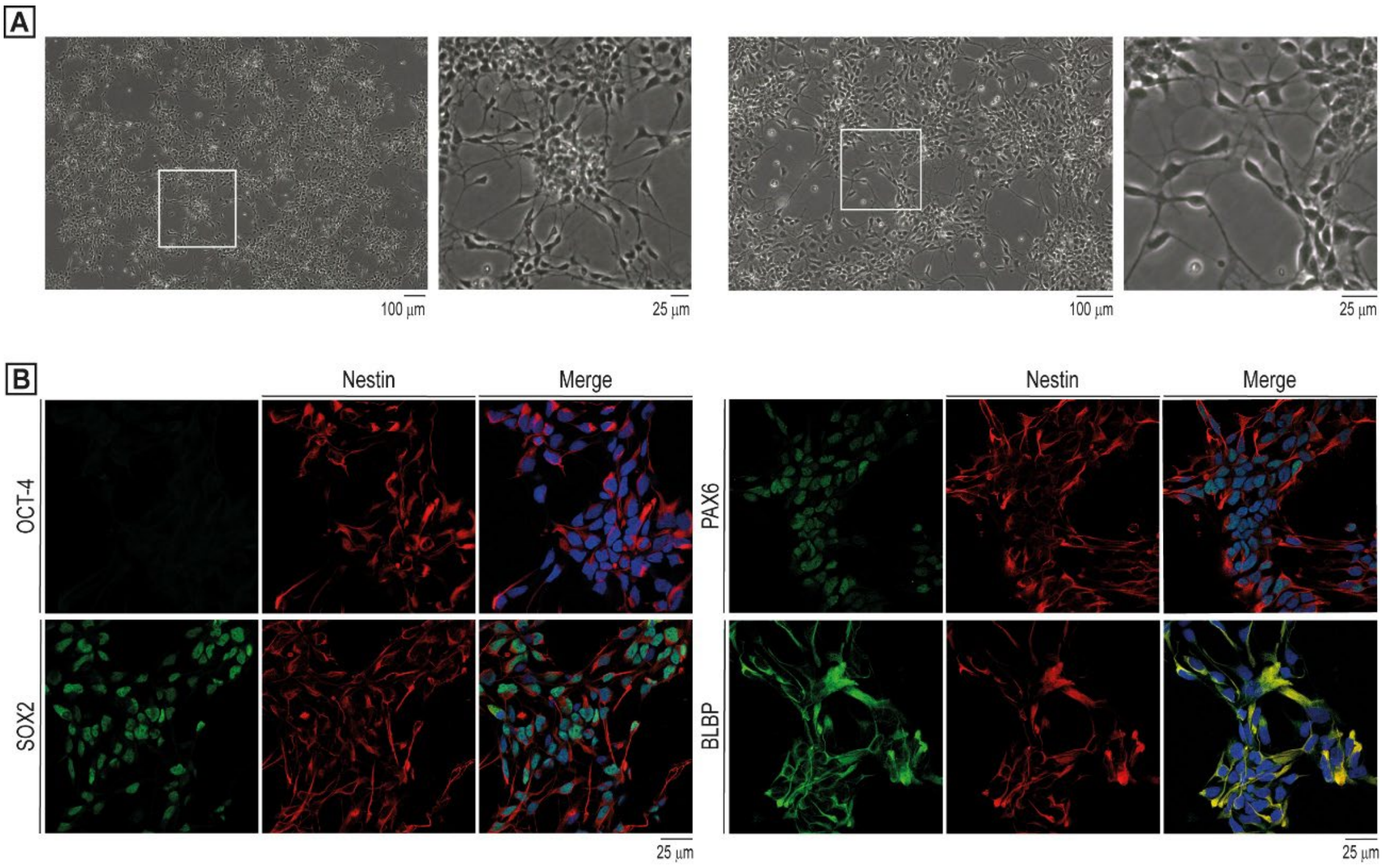

Figure S1. Characterization of human iPSC-derived neural stem cells (NSCs). (A) Phase-contrast micrographs showing the typical rosette-like architecture of NSC cultures at passage 16 (DCC1 line). White boxes are the regions magnified in the adjacent insets. Scale bars: 100 µm (main fields) and 25 µm (insets). (B) Immunofluorescence images demonstrating loss of pluripotency marker OCT-4 and expression of neural progenitor markers SOX2, PAX6, and BLBP (green). Nestin (red) and nuclei (blue, DAPI) are shown in merged channels for reference. Scale bar: 25 µm.

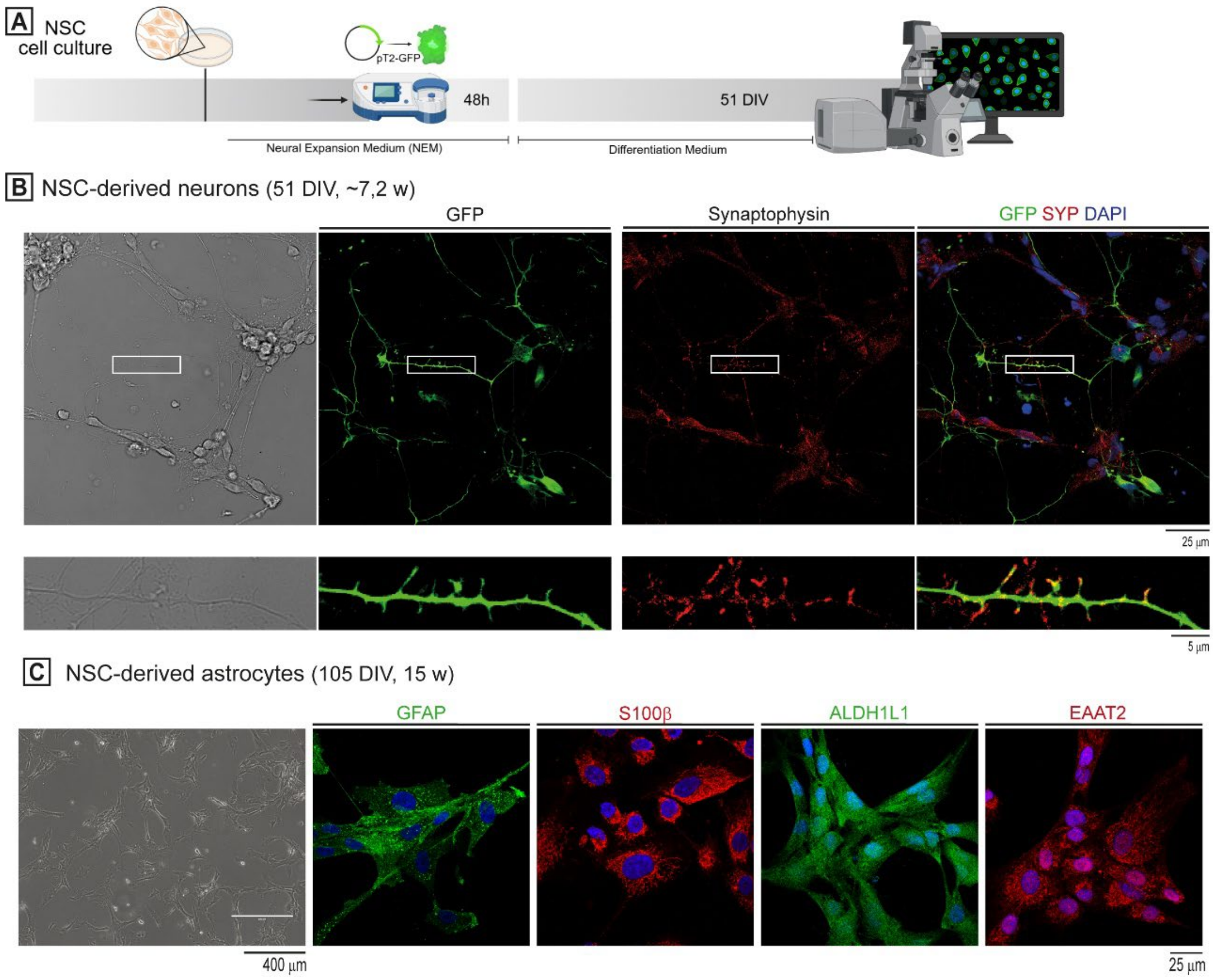

Figure S2. Differentiation capacity of human NSCs toward neuronal and astroglial lineages. (A) Schematic illustration of NSCs electroporated with a GFP expression vector for long-term culture under a neuronal differentiation medium. (B) Representative images of GFP^+ cells (CF2 line) after 51 days of differentiation immunolabeled for synaptophysin (SYP, red), a presynaptic terminal marker, and nuclei are counterstained with DAPI (blue). Higher-magnification views of the indicated regions highlight synaptophysin puncta along GFP^+ neuronal processes. The lower image shows an enlarged view of the area outlined by the white rectangle above. Scale bars: 25 µm (field) and 5 µm (magnified views). (C) Representative (CF1 line) brightfield image illustrates the overall cell morphology after 15 weeks of astroglial differentiation. Scale bar: 400 µm. Immunofluorescence images (CF2 line) at 15 weeks show expression of astrocyte markers displayed as merged images with nuclear counterstaining (DAPI, blue): glial fibrillary acidic protein (GFAP; green), S100 calcium-binding protein B (S100β; red), aldehyde dehydrogenase 1 family member L1 (ALDH1L1; green), and excitatory amino acid transporter 2 (EAAT2; red). Scale bar: 25 µm.

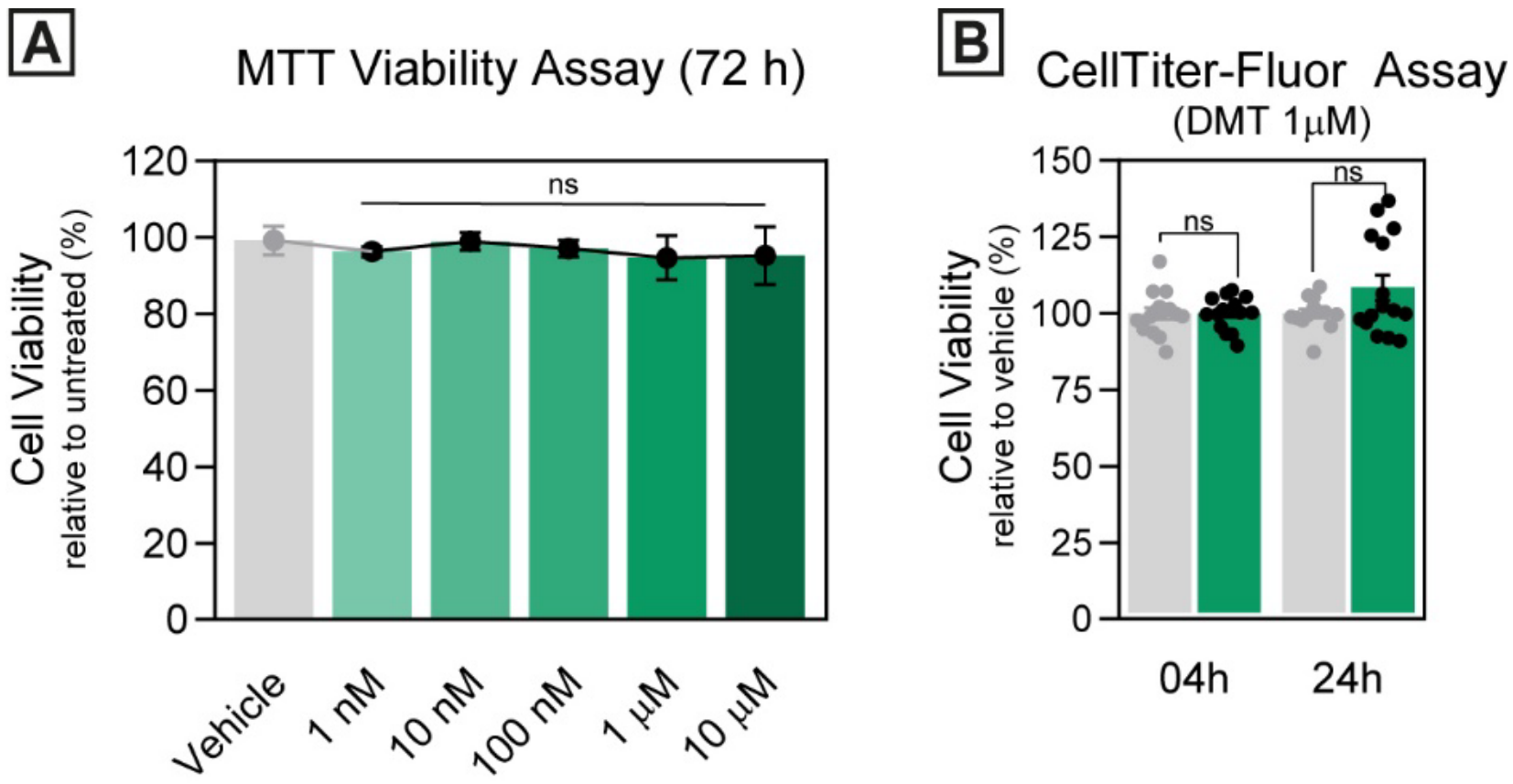

Figure S3. DMT does not impair NSC viability. (A) Cell viability assessed by MTT assay after 72 h exposure to DMT (1 nM–10 µM), showing no significant differences relative to vehicle (mean ± SEM, N = 3, Mixed-effects, Dunnett's post hoc; *ns* = nonsignificant, $p > 0.05$). Consistent across 3 iPSC-derived NSC lines tested. (B) Cell viability measured by CellTiter-Fluor assay following acute DMT exposure (1 µM, green bars) for 4 h and 24 h, presented side by side and normalized to vehicle controls (gray bars), with no detectable reduction in viability at either time point (mean ± SEM, N = 3, Mixed-effects, Sidak's post hoc, *ns* = non significant, $p > 0.05$). Consistent across 2 iPSC-derived NSC lines tested.

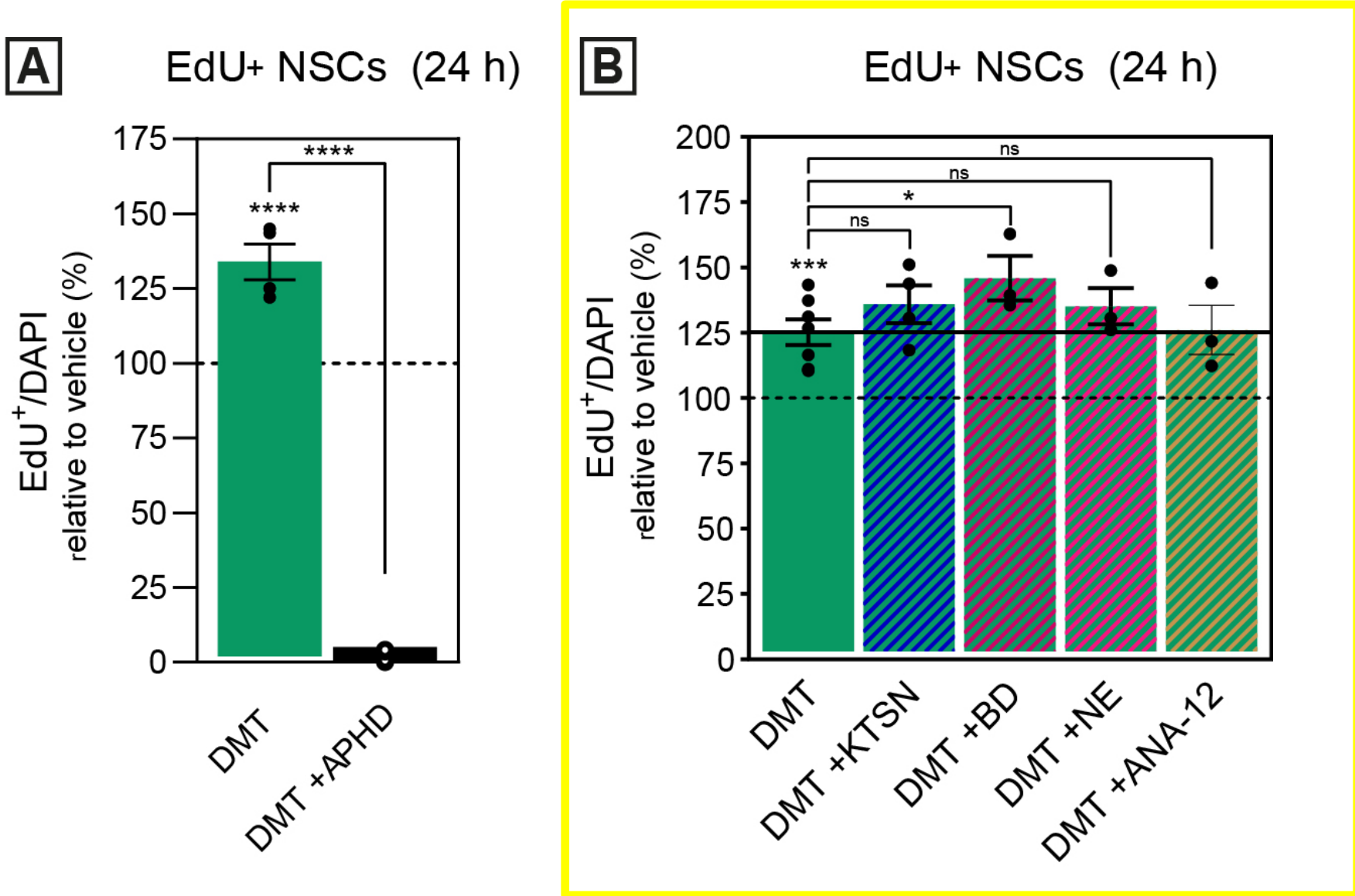

Figure S4. EdU incorporation reflects bona fide DNA synthesis but none of the antagonists evaluated prevented the DMT-induced increase. (A) Validation of bona fide DNA synthesis in the EdU assay. Co-treatment with the DNA polymerase inhibitor aphidicolin (APHD; 1 µM) abolished EdU incorporation relative to both vehicle and DMT (1 µM), confirming that the EdU signal reflects active DNA replication (mean ± SEM, N = 4, Mixed-effects, Dunnett's post hoc; $^{****}p < 0.0001$). Consistent across 3 iPSC-derived NSC lines tested. (B) Pharmacological interrogation of DMT-induced EdU incorporation using single-dose antagonism of 5-HT2A (ketanserin, KTSN), σ1R (BD-1063, BD; or NE-100, NE), and TrkB (ANA-12). None of the antagonists prevented the DMT-induced increase in EdU incorporation under these assay conditions. Consistent across 2 iPSC-derived NSC lines tested. Data are shown as mean ± SEM; N = 3, Mixed-effects, Dunnett's post hoc; $^{*}p < 0.05$.

Supplementary tables

Table S1. Resources and reagents

Resource	Source	Identifier	Notes
Human iPSC cell lines			
Healthy_female_Control1	Coriell Institute	GM23279A, RRID:CVCL_F178	Karyotype 46, XX, Age 36
Healthy_female_Control2	IDOR	DCC1	Karyotype 46, XX, Age 18
Healthy_male_Control1	IDOR	CF1	Karyotype 46, XY, Age 37
Healthy_male_Control2	IDOR	CF2	Karyotype 46, XY, Age 31
Healthy_male_Control3	IDOR	ADHD2	Karyotype 46, XY, Age 31
Media and supplements			
Advanced DMEM	Thermo Scientific	Cat# 12634010	
B-27™ Supplement	Gibco	Cat# 17504044	
DMEM/F12 (1:1)	Gibco	Cat# 11330032	
Fetal bovine serum (FBS)	Gibco	Cat# 10270106	Astrocyte Induction Medium
Fetal bovine serum (FBS)	VWR	Cat# 89510194	FACS Buffer
N-2 Supplement	Gibco	Cat# 17502001	
Neurobasal™ Medium	Gibco	Cat# 12348017	
PSC Neural Induction Medium	Gibco	Cat# A1647701	Includes Neural Induction Supplement (NIS)
Chemicals and drugs			
N,N-DMT	Usona Institute	CAS# 61-50-7	
ANA 12	Tocris Bioscience	Cat# 4781	
Aphidicolin	Tocris Bioscience	Cat# 5736	
BD 1063 dihydrochloride	Sigma-Aldrich	Cat# SML027	
Ketanserin (+)-tartrate salt	Sigma-Aldrich	Cat# S006	
Laminin	Gibco	Cat# 23017-015	
Palbociclib (PD-0332991) HCl	Selleckchem	Cat# S1116	
Poly-L-ornithine	Sigma-Aldrich	Cat# P3655	
Triton™ X-100	Sigma-Aldrich	Cat# T8787	
Y-27632	Sigma-Aldrich	Cat# 688000	
NE-100	Tocris Bioscience	Cat# 3133	
Assay kits			
CellTiter-Fluor™ Cell Viability	Promega	Cat# G6080	
CellTiter-Glo® 3D Cell Viability	Promega	Cat# G9681	
Click-iT™ EdU Kit for Imaging	Invitrogen	Cat# C10337	
Lumit® p-RB (Ser807/811) ICS	Promega	Cat# W1331	
MycoAlert™	Lonza	Cat# LT07-418	
Total BDNF Quantikine ELISA Kit	R&D Systems	Cat# DBNT00	
Buffers and enzymatic reagents			
Accutase	Sigma-Aldrich	Cat# A6964	
Accutase	Gibco	Cat# 14190-144	
Cytofix/Cytoperm™ Kit	BD Biosciences	Cat# 554714	

Resource	Source	Identifier	Notes
DNase I Amplification Grade	Invitrogen	Cat# 18068015	
GoTaq® qPCR Master Mix	Promega	Cat# A6001	
M-MLV Reverse Transcriptase	Invitrogen	Cat# 28025013	
M-MLV Reverse Transcriptase	Promega	Cat# M1701	
Pierce™ BCA Protein Assay	Thermo Scientific	Cat# 23225	
Protease Inhibitor Cocktail	Sigma-Aldrich	Cat# P2714	
ReliaPrep™ RNA Miniprep System	Promega	Cat# Z6111	
RIPA Lysis and Extraction Buffer	Thermo Scientific	Cat# 89900	
TaqMan™ Array, Human Cyclins & Cell Cycle Regulation	Applied Biosystems	Cat# 4418768	
TaqMan™ Fast Advanced Master Mix	Applied Biosystems	Cat# 4444557	
Tissue-Tek® O.C.T. Compound	PANTek	Cat# 4583	
Software and equipment			
CellProfiler software	Broad Institute	RRID:SCR_007358	v4.2
Cytation™ 5 Cell Imaging Reader	BioTek	RRID:SCR_019732	
FlowJo™ software	BD Biosciences	RRID:SCR_008520	
GloMax® 96 software	Promega	RRID:SCR_026323	
GloMax® Discover	Promega	RRID:SCR_023469	
GloMax® Explorer	Promega	RRID:SCR_018614	
GraphPad Prism software	GraphPad Software	RRID:SCR_002798	v8.0.1
Infinite® 200 PRO	Tecan	RRID:SCR_020543	
LSRFortessa™ Flow Cytometer	BD Biosciences	RRID:SCR_018655	
BD Accuri C6 Plus Flow Cytometer	BD Biosciences	RRID:SCR_019591	
Nucleofector™ II	Lonza	RRID:SCR_022262	
StepOne Software	Applied Biosystems	RRID:SCR_014281	v2.3
StepOnePlus™ Real-Time PCR System	Applied Biosystems	RRID:SCR_015805	
TCS SP8 Confocal Microscope	Leica	RRID:SCR_018169	

Table S2. Description of cell lines used for each experiment

Assay	Figure	n	N	Consistent across cell lines (#)	DCC1 (ref)	GM79A	CF1	CF2	ADHD2
EdU	1C,D	6	5	3	XX	XX		X	
Ki-67	2A	6	5	3	XX	X		XX	
qPCR	2B	3	4	2	XXX	X			
pRb	2C	5	3	2	XX		X		
qPCR	2D	3	4	2	XXX	X			
ELISA	2E	3	3	2	X		XX		
NP size	3C	10 NPs	4	2	XXX	X			
CTG-3D	3D	5 NPs	7	3	XXXX	X			XX
IHC Nestin	4B	15 NPs	4	2	XXX	X			
IHC TUBB3	4B	15 NPs	4	2	XXX	X			
IHC MAP2	4B	15 NPs	3	2	XX	X			
Flow Nestin+	4C	5 NPs	7	3	XXX	XX			XX
Flow TUBB3+	4C	5 NPs	5	3	XXX	X			X
Flow MAP2+	4C	5 NPs	7	3	XXX	XX			XX
MTT	S3A	6	3	3	X	X		X	
CTG-Fluor	S3B	5	3	2	XX		X		
EdU	S4A	4	3	3	XX	X		X	
EdU	S4B	6	3	2	XX	X			

n = average of replicates (wells/images) or individual neurospheres (NPs) per experiment per condition.
N = independent experiments treated as biological units for statistical inference (experiment-level means).

Table S3. Antibodies and dilutions

Antibody target (host)	Source	Identifiers	Working dilution (and application)
Nestin (rabbit)	Neuromics	Cat# RA22125	1:500 (ICC); 1:750 (IHC); 1:100 (FC)
Oct-4 clone 10H11.2 (mouse)	Millipore	Cat# MAB4401	1:100 (ICC)
PAX6 (rabbit)	Invitrogen	Cat# 42-6600	1:200 (ICC)
SOX2 (rabbit)	Invitrogen	Cat# PA1-094	1:100 (ICC)
BLBP / FABP7 (rabbit)	Abcam	Cat# ab110099	1:100 (ICC)
Synaptophysin (mouse)	Neuromics	Cat# MO20000	1:100 (ICC)
GFAP (mouse)	Neuromics	Cat#MO15052	1:100 (ICC)
ALDH1L1 (rabbit)	Abcam	Cat# AB190298	1:500 (ICC)
S100 beta [EP1576Y] (rabbit)	Abcam	Cat# AB52642	1:100 (ICC)
EAAT2 (rabbit)	Abcam	Cat# ab41621	1:100 (ICC)
Ki-67 (mouse)	BD Pharmingen	Cat# 550609	1:50 (ICC)
βIII-tubulin / TUBB3 clone TUJ1 (chicken)	Aves	Cat# TUJ8847981	1:100 (IHC)
βIII-tubulin / TUBB3 clone TUJ1 (mouse)	Stem Cell Technologies	Cat# 60052	1:100 (FC)
MAP2 or Microtubule associated protein 2 clone AP-20 (mouse)	Sigma-Aldrich	Cat# M1406	1:500 (IHC)
MAP2 or Microtubule associated protein 2	Neuromics	Cat# MO22116	1:100 (FC)
Phospho-Rb (Ser807/811) (D20B12)	Cell Signaling	Cat# 8516	
Rb (4H1)	Cell Signaling	Cat# 9309	
Anti-rabbit IgG (H+L), Alexa Fluor 488 (goat)	Invitrogen	Cat# A11008	1:400 (ICC, IHC), 1:1000 (FC)
Anti-mouse IgG (H+L), Alexa Fluor® 488	Invitrogen	Cat# A11001	1:400 (ICC, IHC)
Anti-rabbit IgG (H+L), Alexa Fluor® 594	Invitrogen	Cat# A11037	1:400 (ICC, IHC)
Anti-mouse IgG (H+L), Alexa Fluor® 594 (goat)	Invitrogen	Cat# A11032	1:400 (ICC, IHC)
Anti-mouse IgG (H+L), Alexa Fluor™ Plus 647 (donkey)	Invitrogen	Cat# A32787	1:1000 (FC)
Anti-Chicken IgY (H+L), Alexa Fluor® 633 (goat)			
DAPI	Roche	10236276001	N / A

*ICC, immunocytochemistry; IHC, immunohistochemistry; FC, flow cytometry.

Table S4. Primers and probes

Target	Target identifier	Source	Custom Primers / Assay_ID
EGF	HGNC:3229	Thermo Scientific	F: ATCAGCTAACCCATTATGGCAAC<br>R: AAAGAAAAGATACTGTGCATCGAC
FGF2	HGNC:3676	Thermo Scientific	F: AGTGTGTGCTAACCGTTACCT<br>R: ACTGCCCAGTTCGTTTCAGTG
GAPDH	HGNC:4141	Thermo Scientific	F: AGCAGGGAGTCCGTAAACG<br>R: AGCATTCCGAAACAGGTAACTTT
HPRT1	HGNC:5157	Thermo Scientific	F: CGTCGTGATTAGTGATGATGAACC<br>R: AGAGGGCTACAATGTGATGGC
BDNF	HGNC:1033	Thermo Scientific	Hs00380947_m1
NGF	HGNC:7808	Thermo Scientific	Hs00171458_m1
NTF3	HGNC:8023	Thermo Scientific	Hs01548350_m1
NTF4	HGNC:8024	Thermo Scientific	Hs01596132_m1
GAPDH	HGNC:4141	Thermo Scientific	Hs99999905_m1
HPRT1	HGNC:5157	Thermo Scientific	Hs02800695_m1

*F, forward; R, reverse.

References

(1) Casas, B. S.; Vitória, G.; do Costa, M. N.; Madeiro da Costa, R.; Trindade, P.; Maciel, R.; Navarrete, N.; Rehen, S. K.; Palma, V. HiPSC-Derived Neural Stem Cells from Patients with Schizophrenia Induce an Impaired Angiogenesis. *Transl. Psychiatry* **2018**, *8* (1), 48. https://doi.org/10.1038/s41398-018-0095-9.

(2) Trindade, P.; Nascimento, J. M.; Casas, B. S.; Monteverde, T.; Gasparotto, J.; Ribeiro, C. T.; Devalle, S.; Sauma, D.; Moreira, J. C. F.; Gelain, D. P.; Porciuncula, L. O.; Palma, V.; Martins-de-Souza, D.; Rehen, S. K. Induced Pluripotent Stem Cell-Derived Astrocytes from Patients with Schizophrenia Exhibit an Inflammatory Phenotype That Affects Vascularization. *Mol. Psychiatry* **2023**, *28* (2). https://doi.org/10.1038/S41380-022-01830-1.

(3) Trindade, P.; Loiola, E. C.; Gasparotto, J.; Ribeiro, C. T.; Cardozo, P. L.; Devalle, S.; Salerno, J. A.; Ornelas, I. M.; Ledur, P. F.; Ribeiro, F. M.; Ventura, A. L. M.; Moreira, J. C. F.; Gelain, D. P.; Porciúncula, L. O.; Rehen, S. K. Short and Long TNF-Alpha Exposure Recapitulates Canonical Astrogliosis Events in Human-Induced Pluripotent Stem Cells-Derived Astrocytes. *Glia* **2020**, *68* (7), 1396–1409. https://doi.org/10.1002/GLIA.23786.

(4) Ledur, P. F.; Karmirian, K.; Pedrosa, C. S. G.; Souza, L. R. Q.; Assis-de-Lemos, G.; Martins, T. M.; Ferreira, J. C. C. G.; de Azevedo Reis, G. F.; Silva, E. S.; Silva, D.; Salerno, J. A.; Ornelas, I. M.; Devalle, S.; Madeiro da Costa, R. F.; Goto-Silva, L.; Higa, L. M.; Melo, A.; Tanuri, A.; Chimelli, L.; Murata, M. M.; Garcez, P. P.; Filippi-Chiela, E. C.; Galina, A.; Borges, H. L.; Rehen, S. K. Zika Virus Infection Leads to Mitochondrial Failure, Oxidative Stress and DNA Damage in Human IPSC-Derived Astrocytes. *Sci. Rep.* **2020**, *10* (1). https://doi.org/10.1038/s41598-020-57914-x.

(5) Souza, L. R. Q.; Pedrosa, C. G. da S.; Puig-Pijuan, T.; da Silva dos Santos, C.; Vitória, G.; Delou, J. M. A.; Setti-Perdigão, P.; Higa, L. M.; Tanuri, A.; Rehen, S. K.; Guimarães, M. Z. P. Saxitoxin Potentiates Human Neuronal Cell Death Induced by Zika Virus While Sparing Neural Progenitors and Astrocytes. *Sci. Rep.* **2024**, *14* (1), 22809. https://doi.org/10.1038/s41598-024-73873-z.

(6) Goto-Silva, L.; Martins, M.; Murillo, J. R.; Souza, L. R. Q.; Vitória, G.; Oliveira, J. T.; Nascimento, J. M.; Loiola, E. C.; Nogueira, F. C. S.; Domont, G. B.; Guimarães, M. Z. P.; Tovar-Moll, F.; Rehen, S. K.; Junqueira, M. Quantitative

Profiling of Axonal Guidance Proteins during the Differentiation of Human Neurospheres. *Biochim. Biophys. Acta Proteins Proteom.* **2021**, *1869* (8). https://doi.org/10.1016/J.BBAPAP.2021.140656.

(7) Yan, Y.; Shin, S.; Jha, B. S.; Liu, Q.; Sheng, J.; Li, F.; Zhan, M.; Davis, J.; Bharti, K.; Zeng, X.; Rao, M.; Malik, N.; Vemuri, M. C. Efficient and Rapid Derivation of Primitive Neural Stem Cells and Generation of Brain Subtype Neurons From Human Pluripotent Stem Cells. *Stem Cells Transl. Med.* **2013**, *2* (11), 862–870. https://doi.org/10.5966/sctm.2013-0080.

(8) Pedrosa, C. da S. G.; Goto-Silva, L.; Temerozo, J. R.; Souza, L. R. Q.; Vitória, G.; Ornelas, I. M.; Karmirian, K.; Mendes, M. A.; Gomes, I. C.; Sacramento, C. Q.; Fintelman-Rodrigues, N.; Cardoso Soares, V.; Silva Gomes Dias, S. da; Salerno, J. A.; Puig-Pijuan, T.; Oliveira, J. T.; Aragão, L. G. H. S.; Torquato, T. C. Q.; Veríssimo, C.; Biagi, D.; Cruvinel, E. M.; Dariolli, R.; Furtado, D. R.; Borges, H. L.; Bozza, P. T.; Rehen, S.; Moreno L. Souza, T.; Guimarães, M. Z. P. Non-Permissive SARS-CoV-2 Infection in Human Neurospheres. *Stem Cell Res.* **2021**, *54*, 102436. https://doi.org/10.1016/J.SCR.2021.102436.
